# Genome-Wide Identification of Ampicillin Resistance Determinants in *Enterococcus faecium*

**DOI:** 10.1371/journal.pgen.1002804

**Published:** 2012-06-28

**Authors:** Xinglin Zhang, Fernanda L. Paganelli, Damien Bierschenk, Annemarie Kuipers, Marc J. M. Bonten, Rob J. L. Willems, Willem van Schaik

**Affiliations:** Department of Medical Microbiology, University Medical Center Utrecht, Utrecht, The Netherlands; Uppsala University, Sweden

## Abstract

*Enterococcus faecium* has become a nosocomial pathogen of major importance, causing infections that are difficult to treat owing to its multi-drug resistance. In particular, resistance to the β-lactam antibiotic ampicillin has become ubiquitous among clinical isolates. Mutations in the low-affinity penicillin binding protein PBP5 have previously been shown to be important for ampicillin resistance in *E. faecium*, but the existence of additional resistance determinants has been suggested. Here, we constructed a high-density transposon mutant library in *E. faecium* and developed a transposon mutant tracking approach termed Microarray-based Transposon Mapping (M-TraM), leading to the identification of a compendium of *E. faecium* genes that contribute to ampicillin resistance. These genes are part of the core genome of *E. faecium*, indicating a high potential for *E. faecium* to evolve towards β-lactam resistance. To validate the M-TraM results, we adapted a Cre-*lox* recombination system to construct targeted, markerless mutants in *E. faecium*. We confirmed the role of four genes in ampicillin resistance by the generation of targeted mutants and further characterized these mutants regarding their resistance to lysozyme. The results revealed that *ddcP*, a gene predicted to encode a low-molecular-weight penicillin binding protein with D-alanyl-D-alanine carboxypeptidase activity, was essential for high-level ampicillin resistance. Furthermore, deletion of *ddcP* sensitized *E. faecium* to lysozyme and abolished membrane-associated D,D-carboxypeptidase activity. This study has led to the development of a broadly applicable platform for functional genomic-based studies in *E. faecium*, and it provides a new perspective on the genetic basis of ampicillin resistance in this organism.

## Author Summary

*Enterococcus faecium* has emerged as an important nosocomial pathogen around the world. Clinical *E. faecium* isolates are often resistant to multiple antibiotics, thereby complicating therapeutic interventions. However, the molecular mechanisms that contribute to the recent emergence of *E. faecium* as a nosocomial pathogen of major importance are only poorly understood, which is, at least partially, due to the lack of appropriate genetic tools for the study of this organism. Here, we developed a systematic genome-wide strategy, based on transposon mutagenesis and microarray-based screening, to identify *E. faecium* genes that contribute to ampicillin resistance. We also adapted the Cre-*lox* recombination system to construct targeted, markerless mutants in *E. faecium*. These tools enabled us to perform both high-throughput genome-wide analysis and specific targeted investigations in a clinical *E. faecium* isolate. We comprehensively identified, confirmed, and characterized a compendium of genes affecting the sensitivity to ampicillin in *E. faecium*. The identified intrinsic ampicillin resistance determinants are highly conserved among *E. faecium*, indicating that this organism has a high potential to evolve towards ampicillin resistance. These ampicillin-resistance determinants may serve as targets for the development of novel antimicrobial therapeutics.

## Introduction

Enterococci rank third overall as causative agents of healthcare-associated infections [1], [2]. Up to the late 1980s, *Enterococcus faecalis* was responsible for practically all enterococcal infections, but starting from the 1990s nosocomial infections with *E. faecium* became more frequent. Currently *E. faecium* causes approximately 40% of all enterococcal infections that are acquired during hospital stay [2]–[4]. Clinical isolates of *E. faecium* have rapidly accumulated antibiotic resistance genes, including those for clinically important antibiotics such as ampicillin and vancomycin, which leads to treatment failure and increased mortality rates [2], [5]–[7]. In the USA, nosocomial infections caused by ampicillin-resistant *E. faecium* (ARE) were first detected in the 1980s and the resistance rates were steadily increasing up to 80% of *E. faecium* isolates in the 1990s [8], [9]. Vancomycin-resistant *E. faecium* (VRE) also emerged in the late 1980s and increased rapidly during the 1990s [9], [10]. Currently, VRE is widespread among clinical *E. faecium* strains in North America, but less common in hospital-acquired infections in Europe [11]. Ampicillin resistance has spread much further and it is currently being reported in over 80% of clinical *E. faecium* isolates from all over the world [1], [2] (European Antimicrobial Resistance Surveillance Network: http://www.ecdc.europa.eu/en/activities/surveillance/EARS-Net/Pages/index.aspx). In addition to ARE and VRE, the emergence of *E. faecium* strains that are resistant to new classes of antibiotics is challenging the few remaining therapeutic options [12]–[14]. Thus, the development of new anti-enterococcal agents may become critical for the successful treatment of infections caused by this multi-drug resistant organism in the future.

The intrinsic resistance to β-lactam antibiotics of enterococci was reported 60 years ago, soon after the introduction of penicillin in the early 1940s, when enterococci were found to be considerably less susceptible to β-lactams than streptococci [15]. Mutations in the high-molecular weight class B penicillin-binding protein 5 (PBP5) have been considered the main cause for the resistance to β-lactams in *E. faecium*. Upregulated expression of *pbp5* and/or mutations in the 3′ end of the gene lead to a further reduced susceptibility to ampicillin [16]–[18]. However, several studies have suggested that the high minimum inhibitory concentration (MIC) of ampicillin against *E. faecium* is not exclusively due to the presence of low-affinity PBP5 but also to other genes or mechanisms that remain to be identified [19], [20]. Recently, Mainardi *et al.* [21], [22] showed in a spontaneous mutant that was obtained in the laboratory by selection on agar media containing ampicillin, that the D,D-transpeptidase activity of the PBPs could be bypassed by a β-lactam resistant L,D-transpeptidase (Ldt_fm_) that catalyses the formation of 3→3 cross-links between peptidoglycan side chains instead of the classical 4→3 cross-links. A D,D-carboxypeptidase, termed DdcY, is an important component in the L,D-transpeptidase mediated pathway of peptidoglycan cross-linking. However, the *ddcY* gene is only present in a small proportion of *E. faecium* isolates [23], again suggesting that additional ampicillin resistance determinants in *E. faecium* remained to be identified and characterized.

Genome-wide studies of clinical *E. faecium* isolates have long been hampered by a lack of appropriate genetic tools. In this study, we describe the construction of a high density *mariner* transposon mutant library and the development of a powerful tool for functional genomics, termed Microarray-based Transposon Mapping (M-TraM), in *E. faecium*. By comparing the mutant library following growth in the presence or absence of ampicillin, we identified a compendium of genes affecting the sensitivity to ampicillin. Targeted mutants of the identified genes with predicted roles in cell wall synthesis were generated for further characterization, which resulted in the identification of several intrinsic ampicillin resistance determinants in *E. faecium*. These ampicillin resistance determinants may serve as targets for the development of novel antimicrobial therapeutics.

## Results

### Construction of a high-density transposon mutant library in *E. faecium*

To attempt genome-wide transposon mutagenesis of the *E. faecium* genome, we constructed the transposon delivery plasmid pZXL5. As shown in Figure S1, this plasmid was composed of a Gram-positive thermo-sensitive replicon, a gentamicin resistant *mariner* transposon with two outward-facing T7 promoters, a nisin-inducible *mariner* transposase, a ColE1 replicon and a *cat* gene. The sequence of pZXL5 was determined by Sanger-sequencing of both DNA strands (Baseclear; Leiden, The Netherlands) and was deposited in GenBank (GenBank Accession Number: JQ088279).

**Figure 1:**
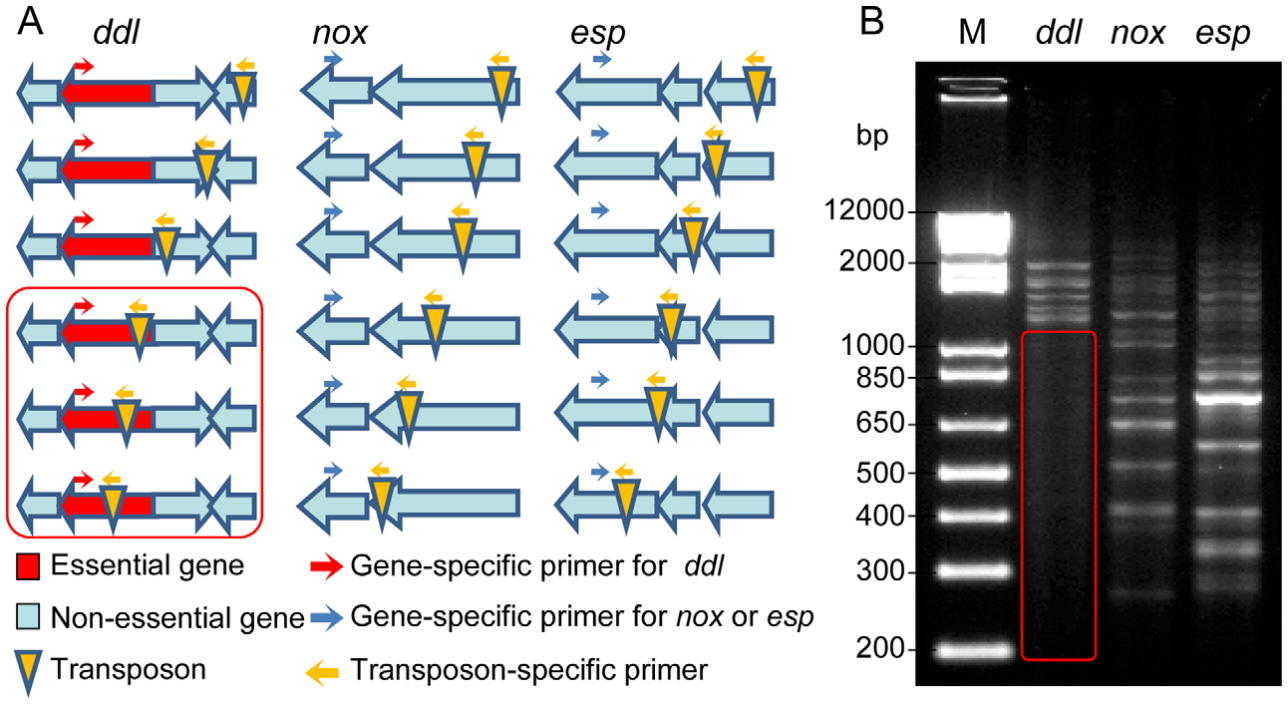
Footprinting analysis of the transposon mutant library. (A) Schematic overview of the transposon footprinting strategy. PCR is performed using a gene specific primer and a primer corresponding to the transposon sequence. (B) Agarose gel electrophoresis of transposon footprinting on the essential gene *ddl* (lane 1), and the non-essential genes *nox* and *esp* (lane 2 and 3, respectively). Each band represents a PCR product of a different size, corresponding to a transposon insertion in a different position. The red box represents the product size range expected for transposon insertions within the essential *ddl* gene.

Using pZXL5, we have produced a transposon mutant library in *E. faecium* strain E1162, an ampicillin-resistant clinical isolate from a bloodstream infection, for which a draft genome sequence has previously been determined [24]. The randomness of the transposon insertions and the absence of multiple transposon insertion events were determined by randomly selecting 17 mutants from the library and carrying out Southern blot hybridizations, using a fragment of the transposon as a probe (Figure S2A), as well as inverse PCR and sequence analysis to determine the location of the transposon insertion point (Figure S2B). The results showed that each mutant carries a single transposon inserted in the genome, and that the transposon was distributed in different loci in the 17 mutants. PCR footprinting was performed to estimate the genome-wide coverage of transposon insertions in the mutant library (Figure 1). An outward-facing primer was designed based on the *mariner* transposon sequence. The other PCR primer was designed for three target genes, *ddl* (which encodes a D-alanine∶D-alanine ligase that is essential for bacterial cell wall biosynthesis [25]), *esp* (which is non-essential and encodes a large surface protein involved in biofilm formation and infection [26]–[28]) and *nox* (which is a non-essential gene encoding a predicted NADH oxidase). Genomic DNA isolated from the pooled mutant library was used as a template. A range of products can be amplified by these primers, each corresponding to a transposon insertion mutant in the library. If a gene is essential for survival, its transposon insertion mutants should not be present in the library after overnight growth, and consequently no PCR products should be amplified in the corresponding size range. As expected, no PCR product was detected within the *ddl* gene (Figure 1) while many PCR bands were found in *esp* and *nox* at intervals of less than 100 bp, indicating the transposon insertions covered the nonessential genes of the genome at high density. Furthermore, mapping the transposon insertion sites to the complete genome sequence of *E. faecium* Aus0004 [29] revealed that transposon insertions were randomly distributed over the genome of this strain and not confined to a specific chromosomal region (data not shown). To establish whether pZXL5 has broad applicability in *E. faecium* we attempted to transform four other clinical *E. faecium* isolates from different geographic origins (Table S1) with pZXL5 using our optimized electroporation protocol. All four strains were efficiently transformed with transformation efficiencies ranging between 110 and 10^5^ transformants per µg DNA (Figure S2C). We then continued to generate transposon mutant libraries in two of these strains (Figure S2D). These observations show that the transposon mutagenesis approach that we initially developed for strain E1162, can also be used for functional genomics in other clinical *E. faecium* isolates.

### M-TraM is highly reproducible

**Figure 2:**
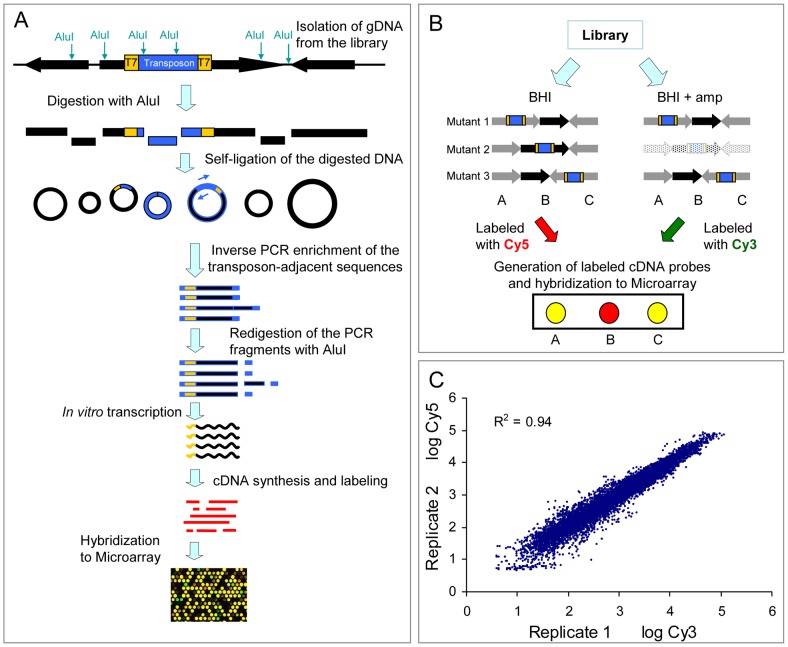
Schematic diagram and reproducibility of M-TraM. (A) Schematic overview of the M-TraM screening. In yellow: inverted terminal repeats (ITRs) of the *himar1* transposon with outward-facing T7 promoters; in blue: the gentamicin resistance gene in the transposon. Genomic DNA is isolated from the *E. faecium* mutant library. DNA is digested with the restriction enzyme *Alu*I, and the DNA fragments are circularized by self-ligation. The transposon-chromosome junction together with an ITR and a T7 promoter is amplified by PCR with primers (blue arrow) that hybridize to the transposon. To eliminate foreign DNA fragments that ligated into the circularized DNA of transposon-chromosome junctions, the PCR products were re-digested with *Alu*I. The purified DNA fragments are used as template in the *in vitro* transcription reaction. The resulting RNA products are reverse transcribed into cDNA. After labelling, the cDNA is used for microarray hybridization. (B) Schematic overview of the screening strategy to identify conditionally essential genes by M-TraM. A chromosomal region encompassing three genes (A, B, and C) from three different mutants (1, 2, and 3) is shown. Each mutant carries a single transposon insertion (blue) that disrupts the function of the gene. Mutant libraries are grown in a control condition (*e.g.*, BHI) and a test condition (*e.g.*, in the presence of ampicillin). All the three genes are non-essential for growth in the control condition. Gene B is required only for the test condition, so mutant 2 exhibits attenuated growth or poorer survival only in the test condition, and will consequently be reduced or be entirely lost from this library (indicated by light shading). M-TraM samples are generated from the two conditions, labelled with different dyes, and hybridized to a microarray. The DNA probes of gene A and gene C on the microarray will hybridize to the samples generated from both conditions. However, the cDNA sample of gene B will be present at reduced levels only in the test condition. By comparing the signal intensity from the two conditions for each probe, genes involved in growth or survival of the test condition can be identified. (C) Reproducibility of M-TraM. Log-log plot of the microarray signal intensities from two independent experiments of mutant libraries grown under non-selective conditions in BHI broth.

As described in the Materials and Methods and in Figure 2, we developed a technique to track the presence of all mutants in the library by simultaneously mapping the transposon insertion sites using microarray hybridization. We termed this technique M-TraM for Microarray-based Transposon Mapping. To validate the reproducibility of M-TraM, we independently grew two aliquots containing approximately 10^7^ cells from the library in 20 ml of BHI broth. After 20 hours of culturing at 37°C, genomic DNA was isolated from the two replicate cultures and used for the generation of cDNA. The cDNA samples were labeled with Cy3 and Cy5 respectively and hybridized to a microarray that was designed using the *E. faecium* E1162 genome sequence. The result showed that M-TraM is highly reproducible, with a correlation coefficient of 0.94 between the two independent experiments (Figure 2C).

### Identification of *E. faecium* genes involved in ampicillin resistance by M-TraM

To identify genes required for ampicillin resistance, we grew the pool of mutants in the presence or absence of a subinhibitory concentration (20 µg ml^−1^) of ampicillin, and used M-TraM to determine which mutants were selectively lost during culturing in the presence of ampicillin. Eleven genes belonging to a variety of functional categories were identified to be involved in ampicillin resistance (Table 1). Four genes involved in cell wall biogenesis were identified and we decided to further focus on these genes. The EfmE1162_0447 (*ddcP*) and EfmE1162_1886 (*ldt*_fm_) genes were predicted to encode a D-alanyl-D-alanine carboxypeptidase (D,D-carboxypeptidase, DdcP) and beta-lactam-insensitive peptidoglycan transpeptidase (L,D-transpeptidase, Ldt_fm_), respectively. Previous studies have shown that a different D,D-carboxypeptidase (DdcY) and Ldt_fm_ were able to bypass the D,D-transpeptidase activity of the PBPs by forming 3→3 cross-links instead of the classical 4→3 cross-links thereby conferring resistance to β-lactams [21], [23]. DdcP and DdcY only share 13.7% amino acid identity and have a completely different protein domain architecture as DdcY is a β-lactam-insensitive VanY-type carboxypeptidase [23], [30], while DdcP belongs to the family of low-molecular-weight (LMW) PBPs [30] (Figure S3). The *ddcY* gene is absent from 24 (including E1162) of the 29 *E. faecium* genomes available (on 9 January 2012) at NCBI Genomes. The *ddcP* gene is conserved in all 29 *E. faecium* genomes. EfmE1162_0975 (*pgt*) was predicted to encode a glycosyl transferase group 2 family protein which is 63% identical to GltB, a protein that was proposed to be involved in glycosylation of cell wall teichoic acid in serotype 4b *Listeria monocytogenes* [31]. EfmE1162_2487 (*lytG*) was predicted to encode an exo-glucosaminidase that could be acting as a peptidoglycan hydrolase involved in cell wall lysis, remodeling and cell division [32]. An overview of the protein domain architecture and predicted cellular localization of DdcP, Ldt_fm_, Pgt and LytG is provided in Figure S3. Like the *ddcP* gene, the *ldt*_fm_, *pgt* and *lytG* genes are present in all the 29 *E. faecium* genomes as well, suggesting these genes are part of the *E. faecium* core genome. Notably the *E. faecium* ampicillin resistance determinants *ddcP*, *ldt*_fm_ and *pgt* do not have homologs (defined here by proteins with >30% amino acid identity) in *E. faecalis*. It should be noted that the L,D-transpeptidase from *E. faecalis* that was biochemically characterized by Magnet *et al.*, [33] has not been experimentally linked to β-lactam resistance in *E. faecalis* and is only remotely related (26% amino acid identity) to *ldt*_fm_.

**Table 1:** *E. faecium* genes involved in ampicillin resistance as determined by M-TraM analysis.

LocusTag^a^	Accession code	Gene Name	Annotation	Fold-change^b^	Bayesian P-value
EfmE1162_0447	ZP_06676292	*ddcP*	D-alanyl-D-alanine carboxypeptidase	32.5	4.4×10^−7^
EfmE1162_2490	ZP_06678189		oxidoreductase, Gfo/Idh/MocA family	11.8	8.6×10^−4^
EfmE1162_1886	ZP_06677646	*ldt*_fm_	beta-lactam-insensitive peptidoglycan transpeptidase	11.1	3.0×10^−5^
EfmE1162_0975	ZP_06676820	*pgt*	glycosyl transferase, group 2 family protein	10.6	4.8×10^−5^
EfmE1162_0256	ZP_06676101		metallo-beta-lactamase superfamily protein	10.0	4.8×10^−4^
EfmE1162_2260	ZP_06678020		dihydrodipicolinate synthase	7.3	3.0×10^−4^
EfmE1162_2058	ZP_06677818		hydrolase, alpha/beta hydrolase fold family	7.1	1.7×10^−4^
EfmE1162_0669	ZP_06676514		nitroreductase family protein	6.8	2.8×10^−4^
EfmE1162_1943	ZP_06677703		chromosomal replication initiator protein DnaA	5.5	7.6×10^−4^
EfmE1162_0064	ZP_06675909		ribosomal protein L33	5.2	2.8×10^−4^
EfmE1162_2487	ZP_06678186	*lytG*	Exo-glucosaminidase	−7.5	6.3×10^−4^

a Indicates the gene containing the transposon insertion.

b Indicates the fold-change derived from the ratio of the unselected control library to the ampicillin competitively selected library. *e.g.* the value 32.5 means that the relative quantity of mutants of EfmE1162_0447 in the ampicillin-selected library was 32.5-fold less than in the control library grown without selective pressure. This indicates that mutants in EfmE1162_0447 have a lower relative fitness in the presence of ampicillin than wild type cells. The value of −7.5 for EfmE1162_2487 indicates that mutants in this gene outgrow the other mutants in the ampicillin-selected library by 7.5-fold, indicating that mutants of EfmE1162_2487 have higher relative fitness in the presence of ampicillin.

### Comparison of ampicillin sensitivities of targeted mutants and wild-type E1162

**Figure 3:**
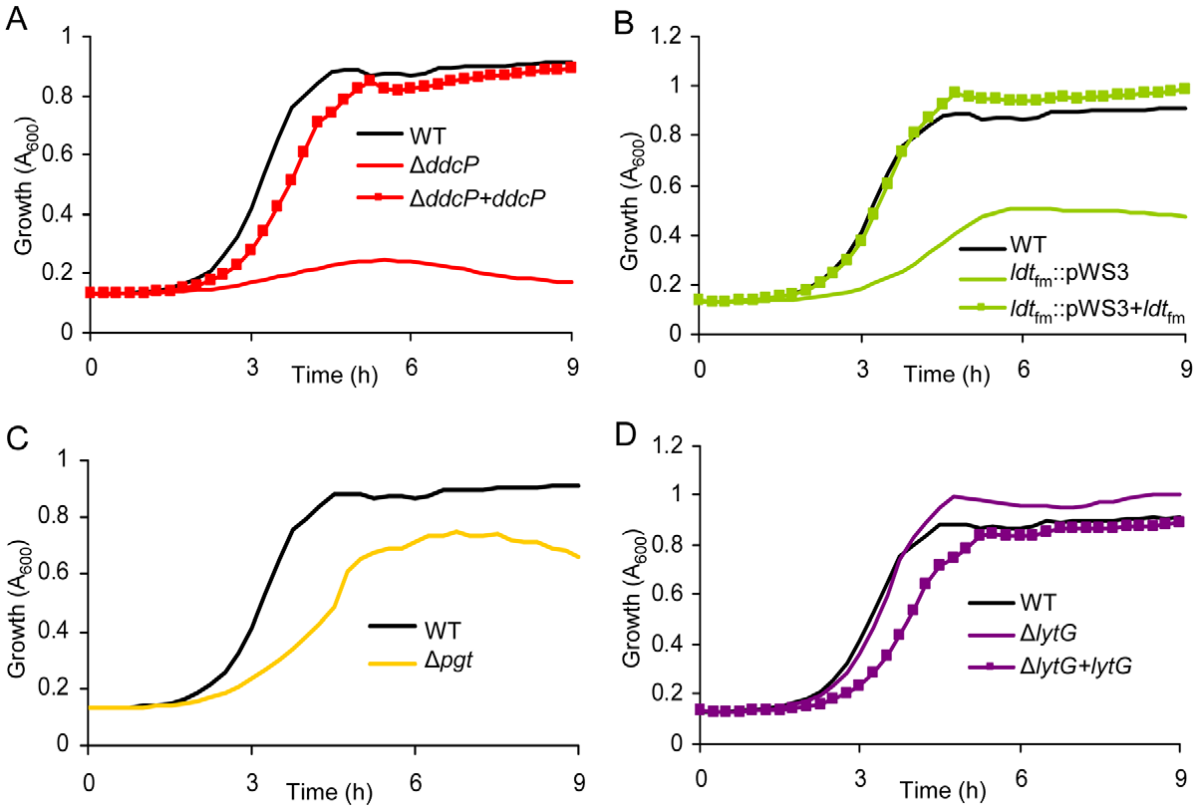
The effect of targeted mutations of *ddcP*, *ldt*_fm_, *pgt*, and *lytG* on growth of *E. faecium* in the presence of ampicillin. Overnight cultures of mutants and wild-type *E. faecium* were inoculated at an initial cell density of OD_660_ 0.0025 in BHI with 20 µg ml^−1^ ampicillin. Growth curves of strain E1162, the different mutants (panel A: Δ*ddcP*; panel B: *ldt*_fm_::pWS3; panel C: Δ*pgt*; panel D: Δ*lytG*) and *in trans* complemented strains are shown. Growth curves are mean data of three independent experiments.

To validate the results of the M-TraM screen and to further characterize the role of the identified genes in ampicillin resistance, we constructed targeted mutants in the *ddcP*, *ldt*_fm_ and *pgt* genes, which were identified with the most significant P-values (Table 1) and have predicted functions in cell wall biogenesis. We also generated a targeted mutant in the *lytG* gene of which the inactivation could confer hyper-resistance to ampicillin, as suggested by the M-TraM data. Targeted deletion mutants of *ddcP*, *pgt*, and *lytG* were generated using a novel Cre-*lox*-based system for the generation of markerless mutants in *E. faecium* that we developed as part of this study (Figure S4). For *ldt*_fm_ no double cross-over mutant could be constructed and instead a single cross-over mutant was constructed using the pWS3 vector [34]. Mutants of *ddcP*, *ldt*_fm_, and *lytG* were also complemented *in trans* and the ampicillin resistance of E1162 (wild-type), the mutants and the complemented strains was determined. The *pgt* mutant could not be complemented as constructs containing the *pgt* gene could not be transformed to either *Escherichia coli* or *E. faecium*, presumably due to toxicity of the gene product. In the absence of ampicillin we did not detect significant differences in growth speed or cell density upon entry into stationary phase between wild-type and the mutant strains (Figure S5). When these strains were grown in BHI with 20 µg ml^−1^ ampicillin, the Δ*ddcP* mutant was dramatically affected in its growth (Figure 3A). Growth of the *ldt*_fm_::pWS3 and Δ*pgt* mutants was also poorer than wild-type (Figure 3B and 3C). The *in trans* complemented strains of the Δ*ddcP* and *ldt*_fm_::pWS3 strains could fully or partially restore the ampicillin resistance to wild-type levels (Figure 3A–3C). The Δ*lytG* mutant had a growth rate that was similar to the parental strain's (0.998±0.007 h^−1^ for Δ*lytG* vs. 0.989±0.018 h^−1^ for E1162) but could grow to slightly higher optical densities (Figure 3D). The *in trans* complemented *lytG* mutant exhibited a significantly lower growth rate (0.859±0.017 h^−1^) in exponential phase (Figure 3D). The empty vector had no effect on the growth of the mutants in BHI supplemented with 20 µg ml^−1^ ampicillin (data not shown). MICs of ampicillin against the wild-type E1162 and Δ*ddcP*, *ldt*_fm_::pWS3, Δ*pgt* and Δ*lytG* strains were determined by microdilution in cation-adjusted Muller-Hinton broth as 43, 8, 16, 27 and 43 µg ml^−1^, respectively, which is in accordance with the growth performance of the mutants in BHI with 20 µg ml^−1^ ampicillin (Figure 3). The seemingly contradictory observation that the *ldt*_fm_::pWS3 mutant can grow in BHI medium supplemented with ampicillin at a concentration above the MIC can be explained by the differences in growth media used and the approximately 4-fold larger inoculum size used in the growth experiments in BHI compared to the inoculum size used in the MIC determinations. The MIC of vancomycin was determined to be 0.5 µg ml^−1^ for all strains.

### Comparative analysis of the transcriptome of *E. faecium* E1162 during exponential growth in the absence and presence of ampicillin

We used microarray-based transcriptome analysis on exponentially growing (OD_660_ = 0.3) *E. faecium* E1162 cultures in BHI medium with or without 20 µg ml^−1^ ampicillin to identify genes that are regulated by the exposure to sub-MIC levels of ampicillin. Compared to the untreated control, only sixteen genes were identified to be differentially regulated between the two conditions and none of these genes were upregulated more than 2.1-fold in the presence of ampicillin (Table S2), indicating that *E. faecium* does not require major transcriptional rearrangements to cope with the presence of sub-inhibitory levels of ampicillin. None of the genes that were identified by M-TraM were identified to be differentially regulated by the presence of ampicillin, which indicates that the identified ampicillin resistance determinants are constitutively expressed, even in the absence of ampicillin.

### Mutations in *ddcP* and *ldt*_fm_ increase sensitivity to lysozyme

**Figure 4:**
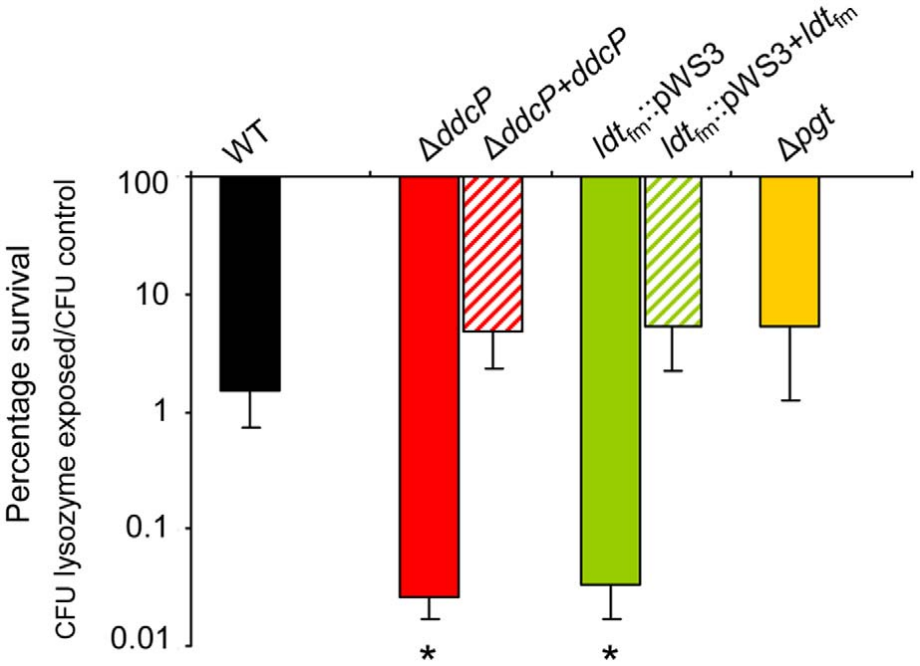
Percentage survival of *E. faecium* cells following a lysozyme challenge. Survival of the indicated wild-type, mutant strains and *in trans* complemented strains following a 30-minute incubation in PBS containing 0.5 mg ml^−1^ lysozyme relative to the survival of the strains after a 30-minute incubation in PBS without lysozyme. Bars represent the standard deviation of the mean of three independent experiments. Asterisks represent significant differences (*P*<0.005 as determined by a two-tailed Student's *t*-test) between the indicated mutants and the wild-type strain.

After the identification of *ddcP*, *ldt*_fm_ and *pgt* as ampicillin resistance determinants, we studied the susceptibility of the wild-type strain E1162 and its mutants to another compound targeting the cell wall, i.e. lysozyme, which is one of the most important antimicrobial enzymes of the host innate immune system. Lysozyme kills Gram-positive bacteria by enzymatic lysis of the bacterial cell wall [35]. The results demonstrated that deletion of *pgt* had no significant effect on lysozyme resistance, while Δ*ddcP* and *ldt*_fm_::pWS3 mutants were significantly more sensitive to lysozyme challenge than the wild-type strain (Figure 4). The *in trans* complemented strains of Δ*ddcP* and *ldt*_fm_::pWS3 could restore the resistance to lysozyme. Hence, *ddcP* and *ldt*_fm_ contribute not only to β-lactam resistance but also to the resistance against the peptidoglycan-hydrolyzing enzyme lysozyme.

### Disruption of *ddcP* abolishes membrane-associated D,D-carboxypeptidase activity of *E. faecium* E1162

**Figure 5:**
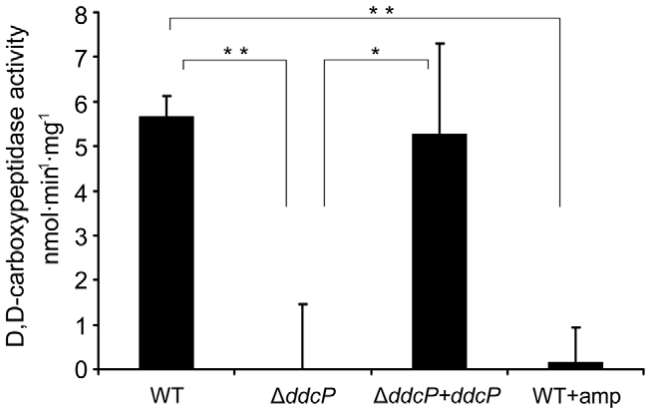
D,D-carboxypeptidase activity in *E. faecium* membrane fractions. Membrane extracts were isolated from the indicated strains grown in BHI medium (with or without the addition 20 µg ml^−1^ ampicillin) until an OD_600_ of 0.7. The D,D-carboxypeptidase activity (nmol min^−1^ mg^−1^) was defined as the number of nmoles of D-Ala released from pentapeptide (7.5 mM) per min and per mg of protein in the membrane fractions. D-Ala was assayed using D-amino acid oxidase coupled to peroxidase. Bars represent the standard deviation of the mean of three independent experiments. Asterisks represent significant difference (^*^ *P*<0.05, ^**^ *P*<0.005 as determined by a two-tailed Student's *t*-test) between the different strains and conditions.

Of all novel ampicillin resistance determinants identified in this study, the *ddcP* gene contributes most to ampicillin resistance in *E. faecium* E1162. The DdcP protein was annotated as a D-alanyl-D-alanine carboxypeptidase but a functional study confirming this activity has not been performed. To confirm its predicted function, we determined the D-alanyl-D-alanine carboxypeptidase activity in cellular extracts of E1162, the Δ*ddcP* mutant and the *in trans* complemented strain Δ*ddcP+ddcP*. As shown in Figure 5, the D-alanyl-D-alanine-carboxypeptidase activity of Δ*ddcP* membrane extracts was completely abolished. In the complemented Δ*ddcP+ddcP* strain enzymatic activity was restored in the membrane fraction, revealing that DdcP is responsible for D-alanyl-D-alanine carboxypeptidase activity in *E. faecium* E1162. The D,D-carboxypeptidase activity was approximately 5 fold lower in the cytoplasmic fractions than in the membrane fractions (data not shown), strongly suggesting that the DdcP protein is associated with the membrane. When *E. faecium* E1162 was grown in the presence of 20 µg ml^−1^ ampicillin, all D-alanyl-D-alanine carboxypeptidase activity was undetectable, which is in accordance with the designation of DdcP as a LMW-PBP.

### Confirmation of *pbp5* as an important ampicillin resistance determinant in *E. faecium* E1162

**Figure 6:**
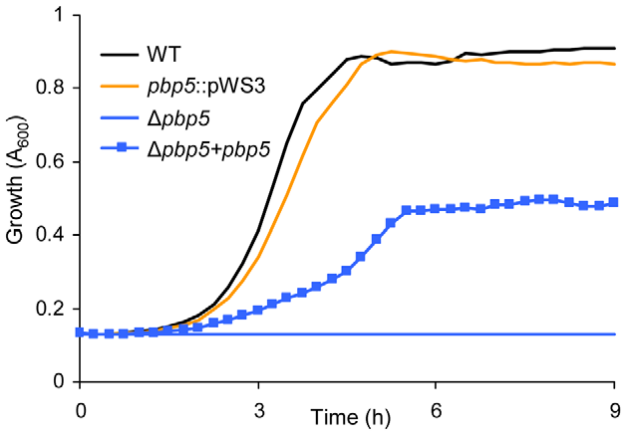
Growth curves of *E. faecium* E1162 and *pbp5* mutants in BHI with 20 µg ml^−1^ ampicillin. Overnight cultures of wild-type *E. faecium* E1162, the insertional *pbp5* mutant (*pbp5*::pWS3), the markerless deletion mutant Δ*pbp5* and the *in trans* complemented deletion mutant (Δ*pbp5*+*pbp5*) were inoculated at an initial cell density of OD_660_ 0.0025 in BHI with 20 µg ml^−1^ ampicillin. Growth curves are mean data of three independent experiments.

Notably, *pbp5* was not identified in this M-TraM screen even though this gene has been implicated in high-level ampicillin resistance in *E. faecium* [16]–[18]. Our failure to identify *pbp5* by M-TraM may partially be explained by the presence of an *Alu*I restriction site on one of the two microarray probes for *pbp5*. The other microarray probe maps to an internal region of the *pbp5* gene and is located on a small *Alu*I restriction fragment with a window of 158 nucleotides for the transposon insertion. Previous genome-wide studies using transposon mutagenesis have shown that transposon insertions might fail to fully inactivate the target genes. The failure is commonly found with insertions situated near either end of a gene, but has also been observed with internal insertions [36], [37], after which genes might be capable of intracistronic complementation. We constructed targeted insertional mutants of *pbp5*, *ddcP* and *ldt*_fm_ by the insertion of the plasmid pWS3 5′ to the gene probes and close to the central region of the gene. Consistent with the M-TraM results and the phenotypes of the markerless mutants, the insertional mutants of *ddcP* (data not shown) and *ldt*_fm_ (Figure 3B) were sensitized to ampicillin. Surprisingly the insertional mutation in *pbp5* did not significantly affect the sensitivity to ampicillin (Figure 6), implying that insertional mutation (by either a plasmid or a transposon) could not fully disrupt *pbp5*. Therefore a markerless deletion mutant for *pbp5* was generated to completely abolish the function of this gene using the Cre-*lox* system described above. To our knowledge, a targeted mutant of the *pbp5* gene has not been generated previously in *E. faecium*. The *pbp5* mutant was unable to grow in cultures containing 20 µg ml^−1^ ampicillin and the ampicillin resistant phenotype could be partially be restored by *in trans* complementation of Δ*pbp5* with the *pbp5* gene (Figure 6). The MIC of ampicillin for Δ*pbp5* was determined by broth microdilution to be only 0.2 µg/ml. Our results revealed that the relative contribution of *pbp5*, *ddcP*, *ldt*_fm_ and *pgt* to ampicillin resistance in *E. faecium* E1162 can be summarized as *pbp5≫ddcp>ldt*_fm_>*pgt*. The *pbp5* mutant was also assayed for its survival in the presence of lysozyme, but no significant difference was found with the parental strain E1162 (data not shown).

## Discussion

Ampicillin resistance in *E. faecium* has emerged in the late 1970s and has spread rapidly since [38]. Practically all clinical isolates are currently resistant to ampicillin. The resistance to β-lactams of *E. faecium* complicates the treatment of infections with this organism, particularly when resistance to other antibiotics has also been acquired. The goal of the research described here was to identify genes involved in ampicillin resistance in *E. faecium* in a high-throughput fashion. We developed a system for the generation of a large random transposon mutant library in *E. faecium*, coupled to a microarray-based screening approach (termed M-TraM) to simultaneously monitor the relative fitness of individual mutants undergoing selection by growth in the presence of ampicillin.

The lack of appropriate genetic tools has long been a bottleneck for the studies of *E. faecium*. In this study, we constructed a random high-density transposon mutant library in *E. faecium*, developed a powerful screening technique to track transposon mutants and adapted the Cre-*lox* [39] recombination system to construct targeted, markerless mutants in *E. faecium*, which enabled us to perform high-throughput genome-wide analysis and specific targeted investigations in a clinical *E. faecium* isolate.

When *E. faecium* is exposed to ampicillin the D,D-transpeptidase activity of PBPs is inhibited, with the exception of the low-affinity PBP5, which can catalyze the last cross-linking step of the D,D-transpeptidation pathway of cell wall assembly [17]. This implies that any D,D-transpeptidase activity conferred by genes other than *pbp5*, is not essential for ampicillin resistance. Consequently, no genes encoding D,D-transpeptidases were identified in this study. However, in our transposon mutant screen, we identified a novel D-alanyl-D-alanine carboxypeptidase (DdcP) that plays an important role in resistance to ampicillin. DdcP is the only gene that is responsible for D-alanyl-D-alanine carboxypeptidase activity during exponential growth of strain E1162. The observation that the enzymatic activity of DdcP is abolished in the presence of ampicillin is in accordance with the prediction that DdcP is a LMW-PBP. The *in vivo* functional roles of LMW-PBPs are relatively poorly understood but they are generally not essential for survival and are thought to contribute to peptidoglycan-remodelling in both Gram-positive and Gram-negative bacteria [40]. Deletion of the gene encoding a LMW-PBP can lead to an increased sensitivity towards β-lactam antibiotics [41]–[42]. Although a mechanistic understanding for this sensitive phenotype is currently lacking, it has been proposed that these LMW-PBPs may enzymatically inactivate β-lactams or, alternatively, save other PBPs from inactivation by sequestration of the β-lactams to the LMW-PBPs [41]. Further functional characterization of DdcP in *E. faecium*, also involving strains that have higher and lower resistance towards ampicillin than strain E1162, is needed to identify the exact mechanism by which DdcP contributes to ampicillin resistance in *E. faecium*. Previous biochemical studies have indicated that Ldt_fm_ is a crucial component of the β-lactam-insensitive L,D-transpeptidation pathway, which catalyzes the cross-links of tetrapeptides [21]–[23]. However, genetic evidence for the role of Ldt_fm_ in ampicillin resistance was so far lacking. Here, we have constructed a mutant, confirming the role of this pathway in ampicillin resistance in *E. faecium*. The identification of *pgt* as an ampicillin resistance determinant in *E. faecium* suggests that wall teichoic acid is involved in β-lactam resistance which is in line with a similar observation in methicillin-resistant *Staphylococcus aureus* (MRSA) strains [43]. None of the mutations had an effect on vancomycin resistance of *E. faecium*, presumably because tetrapeptide precursors for peptidoglycan crosslinks are present at levels that are too low to confer resistance to this antibiotic in wild-type and the mutant strains.

We did not generate mutants or performed functional analyses to confirm the function of the other genes that were identified in our M-TraM screening (Table 1). However, it seems likely that at least some of these genes also contribute to ampicillin resistance in *E. faecium*, in particular EfmE1162_2490 (predicted to function as an NAD- or NADP-dependent oxidoreductase) and EfmE1162_0256 (possibly acting as a zinc-dependent β-lactamase), because the transposon mutants in these genes appear to have a similar loss in fitness in the presence of ampicillin as the transposon mutants in *ldt*_fm_ and *pgt* (Table 1). The *lytG* gene was the only gene that was identified in our screen which, upon inactivation by a transposon, appeared to result in a hyper-resistant phenotype. However, this phenotype could not be confirmed in a markerless deletion mutant of *lytG*, which had the same MIC for ampicillin as the wild-type strain. The observed slightly higher optical density reached by Δ*lytG* as compared to wild-type and the slower growth rate of the *in trans* complemented *lytG* mutant may suggest that there is a subtle role for *lytG* in ampicillin resistance in *E. faecium* but further experiments would be required to exactly determine its role.

The recent emergence of *E. faecium* as a major nosocomial pathogen can be explained by the acquisition of genes that contribute to colonization or infection [26], [44] and by the acquisition of resistance to antibiotics, particularly to ampicillin and vancomycin. Interestingly, resistance against ampicillin and vancomycin emerged predominantly in *E. faecium* while both resistances are virtually absent in *E. faecalis* [8]. This study provides insights into the genetic basis of intrinsic β-lactam resistance in *E. faecium* and identified the ampicillin resistance determinants DdcP, Ldt_fm_ and Pgt in this organism. All three genes are conserved in *E. faecium* but absent from *E. faecalis*, indicating that *E. faecium* possesses more innate β-lactam resistance determinants than *E. faecalis*. This observation supports the concept that *E. faecium* has a higher potential to develop high-level β-lactam resistance than *E. faecalis*, thereby explaining the faster emergence of ampicillin resistance in *E. faecium* than in *E. faecalis* [1], [2], [45].

We have identified several novel mechanisms, besides the low-affinity penicillin-binding protein PBP5, that contribute to ampicillin resistance in *E. faecium*. These proteins could serve as targets for the development of novel therapeutics against this multi-resistant organism. Our study showed that DdcP and Ldt_fm_ also contribute to resistance to lysozyme of *E. faecium*. An inhibitor of these proteins may thus provide the dual benefit of compromising resistance to the innate immune system as well as enhancing antibiotic susceptibility. In the course of this study we have also developed a number of novel genetic tools for *E. faecium* allowing for genome-wide analysis of this bacterium. Further functional genomic-based studies to understand the mechanisms involved in colonization, infection and antibiotic resistance of this important nosocomial pathogen are now a realistic opportunity for future research.

## Materials and Methods

### Bacterial strains, plasmids, and growth conditions

*E. faecium* and *E. coli* strains used in this study are listed in Table S1. The ampicillin-resistant *E. faecium* strain E1162 was used throughout this study. This strain was isolated from a bloodstream infection in France in 1996 and its genome has recently been sequenced [24]. Unless otherwise mentioned, *E. faecium* was grown in brain heart infusion broth (BHI; Oxoid) at 37°C. The *E. coli* strains DH5*α* (Invitrogen) and EC1000 [46] were grown in Luria-Bertani (LB) medium. Where necessary, antibiotics were used at the following concentrations: chloramphenicol 4 µg ml^−1^ for *E. faecium* and 10 µg ml^−1^ for *E. coli*, gentamicin 300 µg ml^−1^ for *E. faecium* and 25 µg ml^−1^ for *E. coli*, spectinomycin 300 µg ml^−1^ for *E. faecium* and 100 µg ml^−1^ for *E. coli*, and erythromycin 50 µg ml^−1^ for *E. faecium* (with added lincomycin at 50 µg ml^−1^) and 150 µg ml^−1^ for *E. coli*. All antibiotics were obtained from Sigma-Aldrich (Saint Louis, MO). Growth was determined by measuring the optical density at 660 nm (OD_660_).

### Construction of an *E. faecium in vivo* transposon mutagenesis system

The transposon delivery plasmid, pZXL5, was constructed in several steps. The Gram-positive *lacZ* gene of pCJK72 [47] was PCR (Accuprime DNA polymerase, Invitrogen) amplified using the primers pCJK72_PstI_lacZ_F and pCJK72_KpnI_lacZ_R (primer sequences are listed in Table S3) and cloned into pTEX5500ts [48] between *Pst*I and *Kpn*I sites to create pZXL1. A fragment containing the chloramphenicol resistance (Chl^r^) cassette, the *lacZ* gene and a gram-positive thermosensitive origin of replication (*repA*ts, functional at 30°C, but not at 37°C) from pZXL1 was PCR amplified using the pZXL1_EcoRI _cm_ori_F and pZXL1_EcoRI _cm_ori_R. Meanwhile, another fragment containing a nisin inducible *mariner* transposase C9 and the *nisRK* genes (encoding a two-component system required for the transcriptional activation of the transposase gene in the presence of nisin), and the ColE1 origin of replication was PCR amplified from pCJK55 [47] using the primers pCJK55_EcoRI_tps_F and pCJK55_EcoRI_tps_R. These two fragments were digested with *EcoR*I and then ligated together to generate pZXL2. To construct a *mariner* transposon [49], the 5′ and 3′ ITRs of *Himar1-mariner* were amplified from pMMOrf [50] using the primer pMMOrf_SacII_ITR that resulted in two *Sac*II recognition sites at both ends of the amplified DNA. The amplified fragment was cloned into pGEM-T Easy (Promega) forming pGEM-ITR. A gentamicin-resistance cassette was PCR amplified from pAT392 [51] using primers pAT392_EheI_T7_genta_F and pAT392_EheI_T7_genta_R, resulting in a gentamicin-resistance cassette with outward-facing T7 promoters on both ends of the cassette, which allows for the generation of RNA products corresponding to the regions flanking the site of transposon integration in genomic DNA. This fragment was digested with EheI and cloned into a SmaI site present between the 5′ and 3′ ITRs in pGEM-ITR, thereby forming the transposon cassette. This transposon cassette was then cut out with *Sac*II and subcloned into the *Sac*II site of pZXL2 producing pZXL3. This vector was electroporated to *E. faecium* E1162 but pZXL3 was found to be able to replicate at 37°C in *E. faecium* E1162 and blue/white screening using *lacZ* proved to be ineffective (data not shown). We therefore replaced the *repA*ts and *lacZ* gene of pZXL3 by the *repA*ts from pAW068 [52]. To this aim, a fragment of pZXL3 containing the nisin inducible *mariner* transposase and the transposon cassette was PCR amplified by primers pZXL3_BfrI_tn_F and pZXL3_BfrI_tn_R. Another fragment carrying the *repA*ts and Chl^r^ cassette was amplified from pAW068 by PCR using primers pAW068_BfrI_cm_ori_F and pAW068_BfrI_cm_ori_R. After digestion with BfrI these two PCR products was ligated together, resulting in the generation of pZXL5. All restriction enzymes were obtained from New England Biolabs.

### Transposon mutant library construction and evaluation

Electrotransformation of the different *E. faecium* strains (Table S1) with the plasmid pZXL5 was performed according to previously described methodologies [26], [34] with optimizations in preparing electrocompetent cells and the cell-plasmid mixture. To obtain the electrocompetent cells, overnight cell culture from BHI was diluted 1000 fold in 25 ml BHI supplemented with 1% of glycine and 200 mM sucrose and again grown overnight at 37°C. Cells were then resuspended in same volume of pre-warmed BHI supplemented with 1% glycine and 200 mM sucrose and incubated at 37°C for 1 hour. Cells were washed three times with ice-cold wash buffer (500 mM of sucrose and 10% glycerol), and resuspended with 1.25 ml ice-cold wash buffer. A 100 µl aliquot of the cell suspension was mixed with 0.1–1 µg of plasmid and transferred into an ice-cooled electroporation cuvette (2-mm gap) and kept on ice for 20 minutes before electroporation. Gentamicin-resistant transformants were grown overnight in BHI broth supplemented with 300 µg ml^−1^ gentamicin and 10 µg ml^−1^ chloramphenicol at the permissive temperature of 28°C, after which 100 µl (approximately 10^8^ viable cells) of this overnight culture were inoculated in 200 ml of pre-warmed BHI broth supplemented with gentamicin and 25 ng ml^−1^ nisin and grown overnight at the non-permissive temperature of 37°C with shaking at 150 rpm. Subsequently, 100 µl of this culture was transferred to 200 ml of fresh pre-warmed BHI broth and similarly grown overnight without nisin. Cultures were then stored at −80°C in BHI broth containing 50% (v/v) glycerol in 1 ml aliquots as mutant library stocks.

To evaluate the randomness and coverage of transposition, we performed Southern blot analysis, identified the sites of transposon insertion and used PCR footprinting. Southern blot analysis was performed as described previously [34]. Genomic DNA of 17 arbitrarily picked gentamicin-resistant colonies from the library was isolated using the Wizard Genomic DNA Purification kit (Promega), digested with HaeIII and BamHI. The probe consisting of a 414 bp fragment within the gentamicin-resistance gene was amplified from pZXL5 by PCR, using the primer pair genta_probe_F/R. To map the sites of transposon insertion, genomic DNA of 17 mutants from the library was digested with HaeIII and then self-ligated, forming circular DNA. Loci in which the transposon had inserted were amplified using the transposon-specific primer pair IPCR_HaeIII_R/F with AccuPrime DNA polymerases (Invitrogen) with the following conditions: 94°C for 1 min; 32 cycles of 94°C for 18 sec, 53°C for 30 sec, 68°C for 10 min; and 68°C for 7 min. Sequencing of the PCR product was performed using the primer IPCR_HaeIII_R and/or IPCR_HaeIII_F. PCR footprinting was conducted on genomic DNA of mutant library as described elsewhere [53] with a transposon-specific primer, ftp_tn, and gene-specific primers, ftp_ddl, ftp_nox or ftp_esp, respectively.

### Simultaneously mapping of transposon insertion sites by M-TraM

Transposon insertion mapping was based on the previously published method of Genomic Array Footprinting (GAF) [54]. Because we observed that T7 polymerase will transcribe *E. faecium* genomic DNA aspecifically (data not shown), it was required to modify GAF to specifically enrich for the junction sites of the transposon and the flanking *E. faecium* DNA. Genomic DNA from mutant libraries was isolated using the Wizard Genomic DNA Purification kit (Promega), digested with *Alu*I (New England Biolabs) and then purified on a Qiagen QIAquick PCR Purification column (Qiagen). 200 ng of the digested DNA was self-ligated by the Quick Ligation Kit (New England Biolabs) in a reaction volume of 20 µl. This ligation reaction was directly used as template for PCR amplification of the transposon–chromosome junctions with primer pair IPCR_*Alu*I_F and IPCR_*Alu*I_R in a reaction volume of 200 µl, using AccuPrime Taq DNA polymerases High Fidelity (Invitrogen) with the following conditions: 94°C for 1 min; 26 cycles of 94°C for 18 sec, 56.5°C for 30 sec, 68°C for 50 sec; and 68°C for 7 min. PCR products were purified using Qiagen QIAquick PCR Purification Kit. After purification, 200–500 ng DNA was redigested by *Alu*I and used for *in vitro* transcription (IVT) in a volume of 20 µl using the T7 MEGAshortscript kit (Ambion) at 37°C for 6 hours. The RNA was first treated with DNase (Ambion) and then purified with the MEGAclear Kit (Ambion). 5–10 µg of the purified RNA was used for generating labeled cDNA using the FairPlay III Microarray Labeling Kit (Agilent Technologies) as described in the manufacturer's protocol. Samples of both conditions (grown in BHI and BHI with 20 µg ml^−1^ ampicillin) were labeled with Cy3 or Cy5. Dyes were swapped between samples to minimize the effect of dye bias. Microarray hybridizations were carried out using the Gene Expression Hybridization Kit (Agilent) following the manufacturer's instructions, using 60 ng of labeled cDNA. The experiment was performed with four biologically independent replicates.

The microarrays used in this study were custom-made *E. faecium* E1162 microarrays using Agilent's 8×15 K platform. Probes were designed by Agilent's eArray server. As probes 60-mer oligonucleotides were designed on coding sequences (CDS) only. A total of 2650 CDS are covered by 2 probes (98.4% of the total number of CDS in the *E. faecium* E1162 genome sequence; NCBI accession number NZ_ABQJ00000000), which were spotted in duplicate. A total of 11 CDS are covered by a single probe (0.4% of the total number of CDS) and these probes were spotted in quadruplicate. For 33 CDS no probes could be designed.

Microarray data were extracted and normalized using Agilent Feature Extraction Software Version 10.7.1.1 (FE 10.7.1). Statistical differences in hybridization signals between the conditions were analyzed using Cyber-T [55] (http://cybert.microarray.ics.uci.edu/). Probes exhibiting Bayesian P-value<0.001 were deemed statistically significant. A gene with two identical probes or all four probes meeting this criterion were classified as significantly selected during exposure to ampicillin.

### Screening for genes involved in ampicillin resistance

To carry out identification of genes required for ampicillin resistance, aliquots containing approximately 10^7^ CFU from the mutant pool stored at −80°C were diluted 1 to 1000 in 20 ml of BHI broth or BHI broth with 20 µg ml^−1^ ampicillin. Cells were grown at 37°C for 8 hours, after which 1 ml of the bacteria cultures were spun down and used for the extraction of genomic DNA, which was then further processed as described above. The same protocol (except that the cells were grown for 20 hours instead of 8 hours) was used to determine the reproducibility of the M-TraM screening in which two independent mutant libraries cultures were mapped using the approach described above.

### Construction of targeted, markerless deletion mutants and *in trans* complementation

For this study, we developed a new method to construct markerless mutants in *E. faecium* based on the Cre-*lox* recombination system [39]. The 5′ and 3′ flanking regions (approximately 500 bp each) of the target genes were PCR amplified with the primers in Table S3. The two flanking regions were then fused together by fusion PCR (generating an *EcoR*I site between both fragments) and cloned into pWS3. Then a gentamicin-resistant cassette was PCR amplified from pAT392 using primers pAT392_EcoRI_lox66_genta_F and pAT392_EcoRI_lox71_genta_R, resulting in a gentamicin-resistant cassette flanked by *lox66* and *lox71*, which allows for the deletion of the gentamicin-resistant cassette in the presence of Cre recombinase. This fragment was digested with *EcoR*I and cloned into the *EcoR*I site that was generated between the 5′ and 3′ flanking regions in the pWS3 construct and then electrotransformed into *E. faecium* as previously described [26], [48]. A transformant containing the plasmid was grown overnight in BHI broth at 30°C supplemented with gentamicin. The cell culture was then diluted 10,000-fold in prewarmed BHI broth and grown at 37°C overnight without antibiotics. The cells were then plated on BHI agar plates with gentamicin and incubated at 37°C. Colonies were then restreaked on BHI agar plates with spectinomycin and BHI agar plates with gentamicin, respectively. The gentamicin-resistant but spectinomycin-susceptible colonies were supposed to be marked deletion mutants and checked by PCR (Table S3). To remove the marker and obtain the markerless mutants a Cre cassette was cut from pRAB1 [56] by digestion with *Pst*I and *Sac*I, blunted by Quick Ligation Kit (New England Biolabs) and cloned into the *EcoR*V site of pWS3 producing pWS3-Cre, which was subsequently electrotransformed into the marked mutants. Spectinomycin-resistant transformants containing pWS3-Cre were then grown overnight in BHI broth at 30°C supplemented with spectinomycin and then diluted 10000 fold in pre-warmed BHI broth and grown at 37°C overnight without antibiotics. These cultures were plated on BHI agar plates and incubated at 37°C for 18–24 h. Single colonies were then restreaked on BHI agar with spectinomycin, BHI agar with gentamicin and BHI agar without antibiotics. The colonies that were susceptible to both gentamicin and spectinomycin resulted from a recombination event catalyzed by Cre and subsequent loss of the thermosensitive plasmid, resulting in a markerless deletion mutant of the gene of interest. This was verified by PCR and sequencing.

Insertional mutagenesis was performed as previously described [34]. Internal DNA fragments of target genes were PCR amplified using primers listed in Table S3, cloned to a Gram-positive thermosensitive plasmid and electrotransformed into *E. faecium* as previously described [26], [48]. After electrotransformation, the cells were recovered for 2 hours at 30°C, after which the cells were plated on BHI plates supplemented with 300 µg ml^−1^ spectinomycin at 30°C to select for transformants. Spectinomycin-resistant colonies were picked and grown overnight in 200 ml of BHI broth at an elevated temperature (37°C) to cure the plasmid. The cells were then plated on BHI agar plates with spectinomycin at 37°C. Single-cross-over integrations into the target genes were verified by PCR with a pWS3-specific primer, check_pWS3, and a gene-specific primer (Table S3).

Plasmids for the *in trans* complementation of the *ddcP*, *ldt*_fm_, *lytG* and *pbp5* mutants were produced by PCR amplification of the genes using the primers listed in Table S3. PCR products were ligated into the downstream region of P*_nisA_* promoter of pMSP3535 [57]. The resulting plasmids were introduced into the appropriate host strains by electroporation as described above.

### Determination of growth curves and MIC

A BioScreen C instrument (Oy Growth Curves AB, Helsinki, Finland) was used to monitor effects of ampicillin on bacterial growth. Wild-type *E. faecium*, mutants and *in trans* complemented strains were grown overnight in BHI and BHI containing appropriate antibiotics. Cells were inoculated at an initial OD_660_ of 0.0025 into 300 µl BHI and BHI with ampicillin 20 µg ml^−1^ and 1 µg ml^−1^.The cultures were incubated in the Bioscreen C system at 37°C with continuous shaking, and absorbance of 600 nm (A_600_) was recorded every 15 min for 9 hours. Each experiment was performed in triplicate.

MIC of ampicillin of the wild-type and mutants were determined in triplicate by broth microdilution in cation-adjusted Muller-Hinton broth as previously described [58].

### Transcriptome profiling

*E. faecium* E1162 was incubated in BHI broth and BHI broth supplemented with 20 µg ml^−1^ ampicillin for 18 hours. Cultures were then diluted to OD_660_ 0.025 in 20 ml of prewarmed BHI broth and BHI broth containing 20 µg ml^−1^ ampicillin respectively, and grown until OD_660_ 0.3. Cells were centrifuged for 12 seconds at 169000 g at room temperature, and pellets were flash frozen in liquid N_2_ prior to RNA extraction. RNA was isolated using TRI Reagent (Ambion) according to the manufacturer's protocol. RNA quantity and quality was determined by spectrophotometry (Nanodrop 1000, Thermo Scientific, Wilmington DE, USA) and by Bioanalyzer 2100 analysis (Agilent). Labeling of 5 µg of total RNA, hybridization and data analysis were performed as described above. Genes for which all four probes exhibited a Bayesian P<0.001 in Cyber-T [55] were deemed differentially expressed.

### Assay for lysozyme sensitivity

To compare the lysozyme sensitivity of the parental strain E1162, the mutant strains and *in trans* complemented strains, overnight cell cultures were diluted 100 fold in fresh BHI and grown to OD_660_ 0.5. Two ml of the cell cultures were harvested by centrifugation. The pellets were resuspended in 1 ml phosphate buffered saline (PBS; NaCl 137 mM; 2.7 mM KCl; 10 mM Na_2_HPO_4_; 2 mM KH_2_PO_4_; pH 7.4) as negative control and in 1 ml PBS containing 0.5 mg ml^−1^ lysozyme. After a 30-minute incubation at 37°C, cells were washed with PBS and resuspended in 1 ml of PBS. Survival of the strains was determined following serial dilution and plating on BHI agar plates. The experiment was performed in triplicate and statistical analysis of the data was performed using a two-tailed Student's *t*-test.

### Determination of D,D-carboxypeptidase activity in enterococcal extracts

The enzymatic activities in the enterococcal extracts of wild-type, Δ*ddcP* and Δ*ddcP+ddcP* were assayed as described previously with slight modifications [59], [60]. In short, strains were grown until an OD_600_ of 0.7. Bacteria were then harvested by centrifugation and lysed by treatment with lysozyme at 37°C for 1 hour followed by sonication. The membrane fraction was then pelleted by ultracentrifugation (100,000 *g*, 45 min). The supernatant (cytoplasmic fractions) was collected and the pellet (membrane fractions) was resuspended in 0.1 M phosphate buffer (pH 7.0) and both fractions were assayed for D,D-carboxypeptidase activity [59], [60]. The amounts of D-Ala released from the pentapeptide (Ala-D-γ-Glu-Lys-D-Ala-D-Ala, Sigma-Aldrich) by D,D-carboxypeptidases were determined by using D-amino acid oxidase and horseradish peroxidase in a colorimetric assay.

### Microarray data accession numbers

The microarray data generated in this study have been deposited in the ArrayExpress database (http://www.ebi.ac.uk/arrayexpress) under accession numbers E-MEXP-3501 for the M-TraM screening for ampicillin resistance determinants, E-MEXP-3502 for the assay of the reproducibility of the M-TraM procedure and E-MEXP-3564 for the transcriptome analysis data.

## Supporting Information

Figure S1 Map of pZXL5. This plasmid contains a Gram-positive thermo-sensitive pWVO1 replicon and the chloramphenicol acetyltransferase (*cat*) gene from pAW068, a nisin inducible *mariner* transposase (including *nisA* promoter, the transposase, *nisK* and *nisR*) and a ColE1 replicon from pCJK55, and a *mariner* transposon carrying the gentamicin resistance gene *acc(6′)-aph(2″)* with two outward-facing T7 promoters. Arrows indicate the direction of transcription. The T7 promoters (P_T7_) and unique or relevant restriction sites are shown.
(PDF)

Figure S2 Evaluation of the transposon mutant library in *E. faecium* E1162 and electroporation of pZXL5 to four other clinical *E. faecium* isolates and subsequent generation of transposon mutant libraries in strains E745 and E1391. (A) Southern blot analysis of 17 randomly selected *E. faecium* transposon insertion mutants (lane 1 to 17) from the mutant library. Genomic DNA was digested with HaeIII and BamHI, and hybridized to an ECL-labeled probe specific for the transposon. (B) Inverse PCR and sequencing analysis of 17 randomly selected *E. faecium* transposon insertion mutants (lane 2 to 18). Genomic DNA was digested with HaeIII, self-ligated and the transposon-chromosome junction was PCR amplified using transposon-specific primers. The PCR products were sequenced and matched to the E1162 genome sequence. The transposon insertion loci of the mutants are indicated. (C) Multiplex PCR verification of the electroporation of pZXL5 into four clinical *E. faecium* strains. The expected sizes of the PCR products of *ddl* (housekeeping gene in the *E. faecium* genome) and the gentamicin resistance cassette on the *mariner* transposon (in pZXL5) are indicated. The primers used for the multiplex PCR are listed in Table S3: ftp_ddl and ddl_1 were used for *ddl*, genta_in_F and genta_in_R were used for pZXL5. (D) Inverse PCR and sequencing analysis of randomly selected transposon insertion mutants from the libraries generated with E745 and E1391. Five mutants were selected from each library. Inverse PCR was performed as described in (B).
(TIF)

Figure S3 Predicted protein domain architecture and cellular localization of Ddcp, Ldt_fm_, Pgt, and LytG. (A) Protein domain visualizations and the annotations of protein domains were made using SMART (Simple Modular Architecture Research Tool) at http://smart.embl.de/. Blue horizontal bars indicate transmembrane regions. Pink stretches indicate regions of low complexity. Pfam domain Peptidase_S11 in DdcP is predicted to function as a serine peptidase with D-Ala-D-Ala carboxypeptidases activity. The PBP5_C domain in DdcP is homologous to the C-terminal domain of *E. coli* low-molecular weight penicillin-binding protein Pbp5, which has no known catalytic function. It could be involved in mediating interactions with other cell wall-synthesizing enzymes, thereby allowing the protein to be recruited to areas of active cell wall synthesis. Alternatively, it could function as a linker domain that positions the active site in the catalytic domain closer to the peptidoglycan layer. The two Pfam PG_binding_4 domains in Ldt_fm_ are predicted to act as a L,D-transpeptidase domain which can cross-link two peptidoglycan side-chains. The Pfam YkuD domain in Ldt_fm_ is frequently encountered in proteins with peptidoglycan-binding domains, but its function is unknown. The Pfam Glycos_transf_2 domain that is present in Pgt is also found in a diverse family of glycosyl transferases that transfer a sugar moiety from an activated nucleotide substrate to a range of substrates including teichoic acids. The Lyz2 domain that was identified in LytG is present in eubacterial enzymes that are distantly related to eukaryotic lysozymes. (B) Cellular localization of the proteins was predicted by Phobius (http://phobius.sbc.su.se/) and PSORTb (http://www.psort.org/psortb/; CM: cytoplasmic membrane; C: cytoplasm; E: extracellular)
(PDF)

Figure S4 Schematic diagram of the Cre-*lox* recombination system for the construction of markerless mutant in *E. faecium*. A detailed description of the procedure is provided in the Materials and Methods section. Step 1: The gene replacement construct carrying *in vitro*-altered sequences (UpFlankingRegion-*lox*66-Gm_r_-*lox*71-DownFlankingRegion) is introduced into E1162 by electroporation, and transformants are incubated at permissive temperature (30°C) for double crossover events. Step 2: The cells are passaged at a non-permissive temperature (37°C) for plasmid replication. Double-crossover integrants, are screened using agar plates supplemented with appropriate antibiotics. Step 3: Subsequently, the thermosensitive plasmid pWS3-Cre is electrotransformed into the marked mutants, and the *lox*66-Gm_r_-*lox*71 cassette is removed from the chromosome by the Cre-mediated excision during overnight culture of the transformants at 30°C. Step 4: Subsequent overnight culturing of the cells at 37°C leads to the loss of pWS3-Cre, resulting in a markerless double crossover mutant in which the gene is replaced by a lox72 site. (Gm^r^: gentamicin resistant; Gm^s^: gentamicin susceptible; Spc^r^: spectinomycin resistant; Spc^s^: spectinomycin susceptible; Ts: thermosensitive replicon).
(TIF)

Figure S5 Growth curves of targeted mutants and wild-type *E. faecium* E1162 in BHI medium without added antibiotics. Overnight cultures of mutants and wild-type strain were inoculated at an initial cell density of OD_660_ 0.0025 in BHI and grown at 37°C with shaking in the Bioscreen C instrument. Growth curves represent mean data from three independent experiments.
(PDF)

Table S1 Strains and plasmids used in this study.
(DOC)

Table S2 Expression ratios of *E. faecium* E1162 genes that exhibit significant differences in expression during mid-exponential growth in BHI and BHI with 20 µg ml^−1^ ampicillin.
(DOCX)

Table S3 Primers used in this study.
(DOC)
