# Supplementary figures and images for: Genome-Wide Identification of Ampicillin Resistance Determinants in Enterococcus faecium
Source: PLoS Genet. 2012 Jun 28;8(6):e1002804. doi: 10.1371/journal.pgen.1002804 (PMC3386183; doi:10.1371/journal.pgen.1002804)

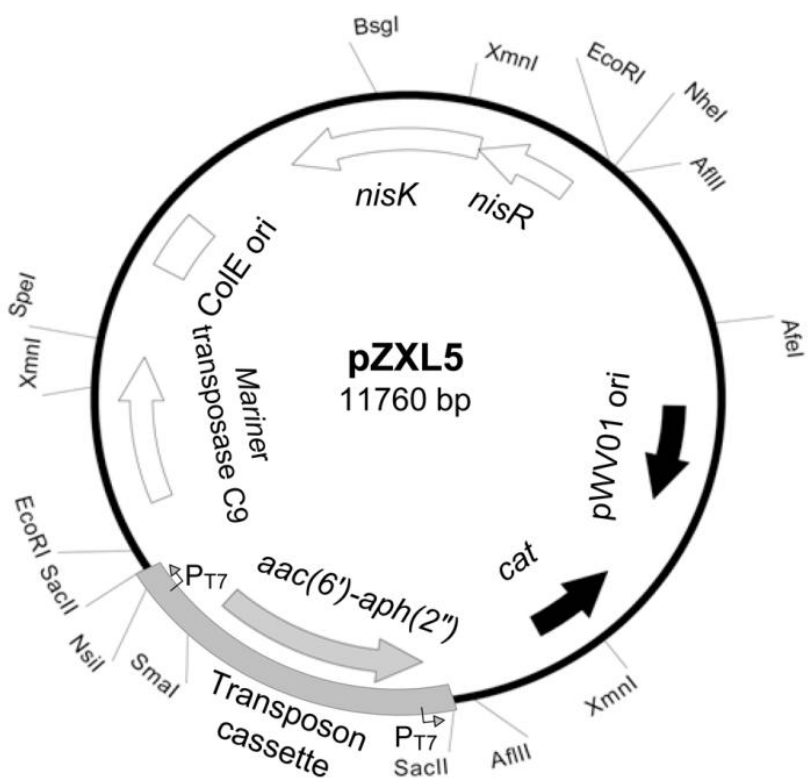

Supplement: Figure S1 — Map of pZXL5. This plasmid contains a Gram-positive thermo-sensitive pWVO1 replicon and the chloramphenicol acetyltransferase (cat) gene from pAW068, a nisin inducible mariner transposase (including nisA promoter, the transposase, nisK and nisR) and a ColE1 replicon from pCJK55, and a mariner transposon carrying the gentamicin resistance gene acc(6′)-aph(2″) with two outward-facing T7 promoters. Arrows indicate the direction of transcription. The T7 promoters (PT7) and unique or relevant restriction sites are shown. (PDF) [file pgen.1002804.s001.pdf]

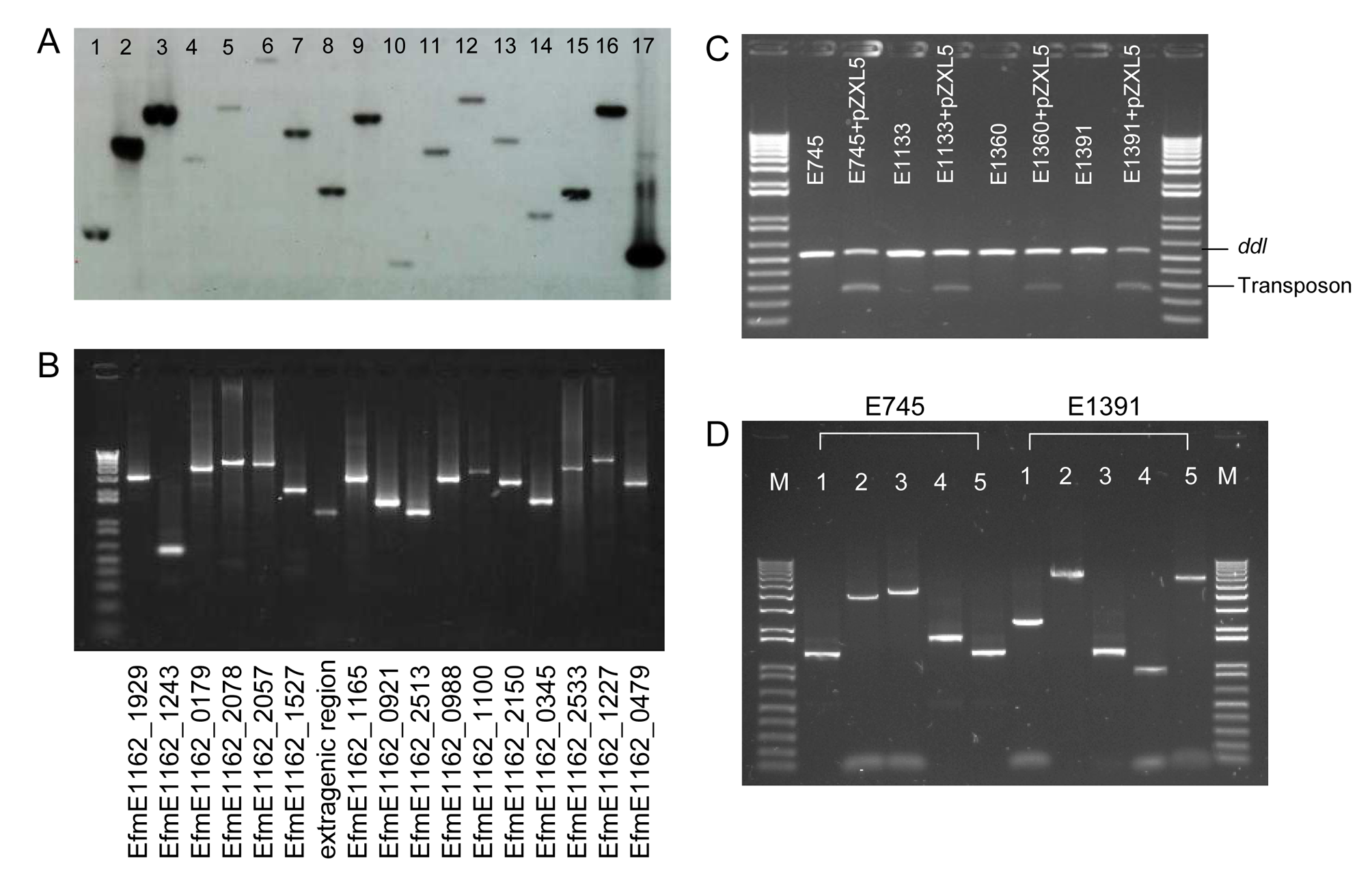

Supplement: Figure S2 — Evaluation of the transposon mutant library in E. faecium E1162 and electroporation of pZXL5 to four other clinical E. faecium isolates and subsequent generation of transposon mutant libraries in strains E745 and E1391. (A) Southern blot analysis of 17 randomly selected E. faecium transposon insertion mutants (lane 1 to 17) from the mutant library. Genomic DNA was digested with HaeIII and BamHI, and hybridized to an ECL-labeled probe specific for the transposon. (B) Inverse PCR and sequencing analysis of 17 randomly selected E. faecium transposon insertion mutants (lane 2 to 18). Genomic DNA was digested with HaeIII, self-ligated and the transposon-chromosome junction was PCR amplified using transposon-specific primers. The PCR products were sequenced and matched to the E1162 genome sequence. The transposon insertion loci of the mutants are indicated. (C) Multiplex PCR verification of the electroporation of pZXL5 into four clinical E. faecium strains. The expected sizes of the PCR products of ddl (housekeeping gene in the E. faecium genome) and the gentamicin resistance cassette on the mariner transposon (in pZXL5) are indicated. The primers used for the multiplex PCR are listed in Table S3: ftp_ddl and ddl_1 were used for ddl, genta_in_F and genta_in_R were used for pZXL5. (D) Inverse PCR and sequencing analysis of randomly selected transposon insertion mutants from the libraries generated with E745 and E1391. Five mutants were selected from each library. Inverse PCR was performed as described in (B). (TIF) [file pgen.1002804.s002.tif]

**A**

Number of amino acids

1 100 200

DdcP

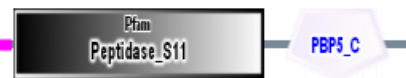

Ldt<sub>fm</sub>

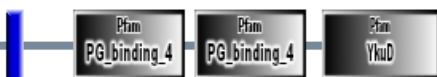

Pgt

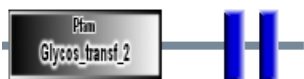

LytG

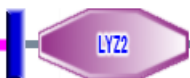

**B**

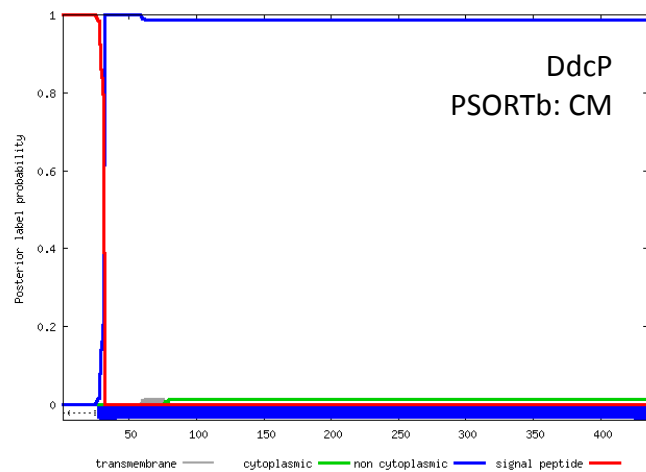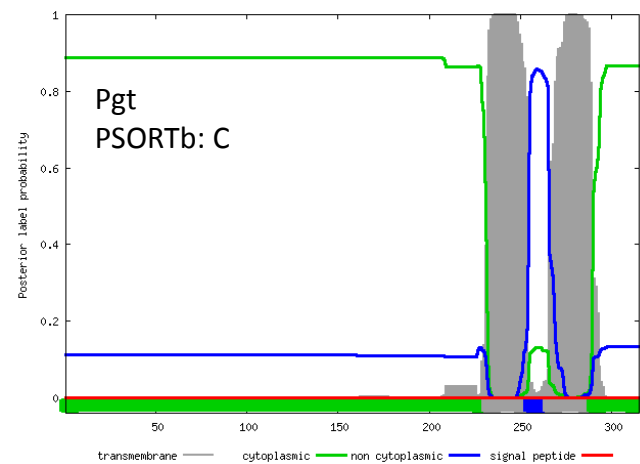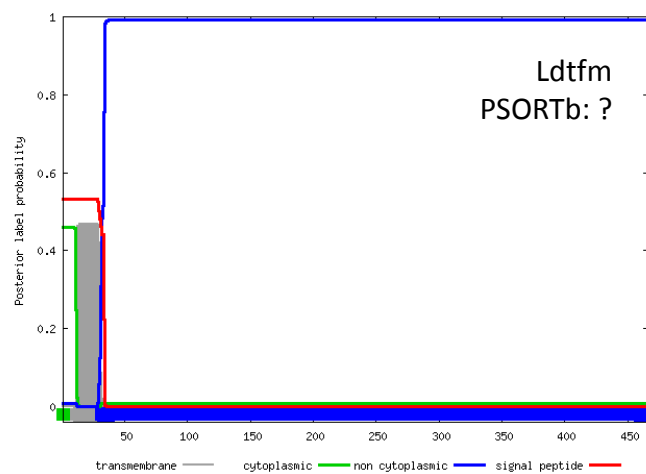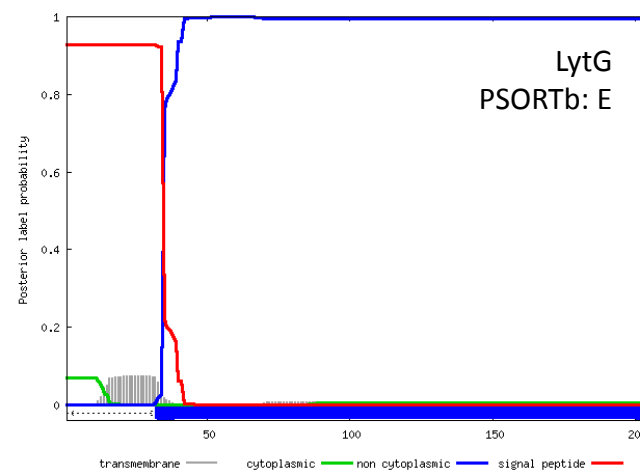

Supplement: Figure S3 — Predicted protein domain architecture and cellular localization of Ddcp, Ldtfm, Pgt, and LytG. (A) Protein domain visualizations and the annotations of protein domains were made using SMART (Simple Modular Architecture Research Tool) at http://smart.embl.de/. Blue horizontal bars indicate transmembrane regions. Pink stretches indicate regions of low complexity. Pfam domain Peptidase_S11 in DdcP is predicted to function as a serine peptidase with D-Ala-D-Ala carboxypeptidases activity. The PBP5_C domain in DdcP is homologous to the C-terminal domain of E. coli low-molecular weight penicillin-binding protein Pbp5, which has no known catalytic function. It could be involved in mediating interactions with other cell wall-synthesizing enzymes, thereby allowing the protein to be recruited to areas of active cell wall synthesis. Alternatively, it could function as a linker domain that positions the active site in the catalytic domain closer to the peptidoglycan layer. The two Pfam PG_binding_4 domains in Ldtfm are predicted to act as a L,D-transpeptidase domain which can cross-link two peptidoglycan side-chains. The Pfam YkuD domain in Ldtfm is frequently encountered in proteins with peptidoglycan-binding domains, but its function is unknown. The Pfam Glycos_transf_2 domain that is present in Pgt is also found in a diverse family of glycosyl transferases that transfer a sugar moiety from an activated nucleotide substrate to a range of substrates including teichoic acids. The Lyz2 domain that was identified in LytG is present in eubacterial enzymes that are distantly related to eukaryotic lysozymes. (B) Cellular localization of the proteins was predicted by Phobius (http://phobius.sbc.su.se/) and PSORTb (http://www.psort.org/psortb/; CM: cytoplasmic membrane; C: cytoplasm; E: extracellular) (PDF) [file pgen.1002804.s003.pdf]

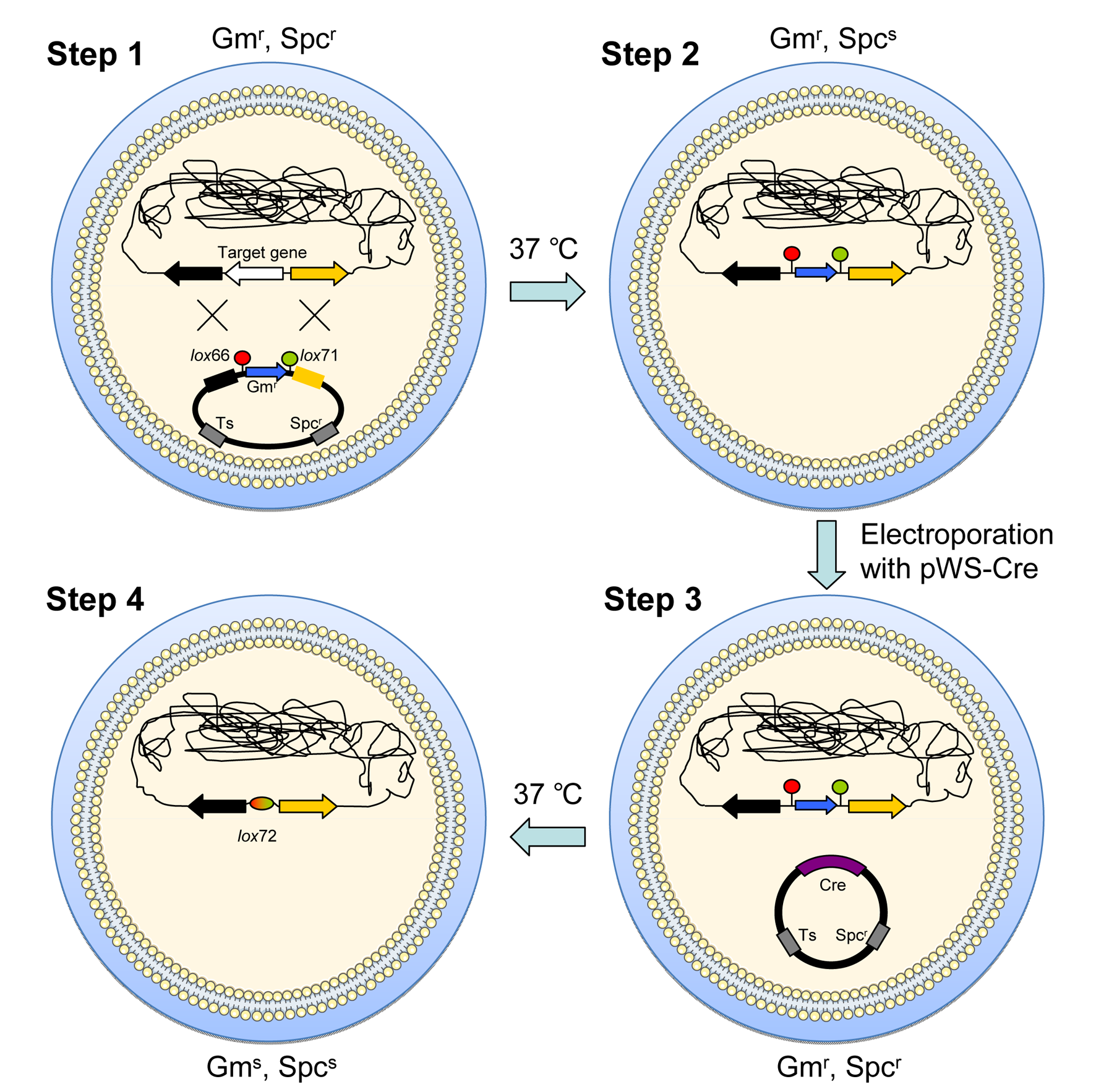

Supplement: Figure S4 — Schematic diagram of the Cre-lox recombination system for the construction of markerless mutant in E. faecium. A detailed description of the procedure is provided in the Materials and Methods section. Step 1: The gene replacement construct carrying in vitro-altered sequences (UpFlankingRegion-lox66-Gmr-lox71-DownFlankingRegion) is introduced into E1162 by electroporation, and transformants are incubated at permissive temperature (30°C) for double crossover events. Step 2: The cells are passaged at a non-permissive temperature (37°C) for plasmid replication. Double-crossover integrants, are screened using agar plates supplemented with appropriate antibiotics. Step 3: Subsequently, the thermosensitive plasmid pWS3-Cre is electrotransformed into the marked mutants, and the lox66-Gmr-lox71 cassette is removed from the chromosome by the Cre-mediated excision during overnight culture of the transformants at 30°C. Step 4: Subsequent overnight culturing of the cells at 37°C leads to the loss of pWS3-Cre, resulting in a markerless double crossover mutant in which the gene is replaced by a lox72 site. (Gmr: gentamicin resistant; Gms: gentamicin susceptible; Spcr: spectinomycin resistant; Spcs: spectinomycin susceptible; Ts: thermosensitive replicon). (TIF) [file pgen.1002804.s004.tif]

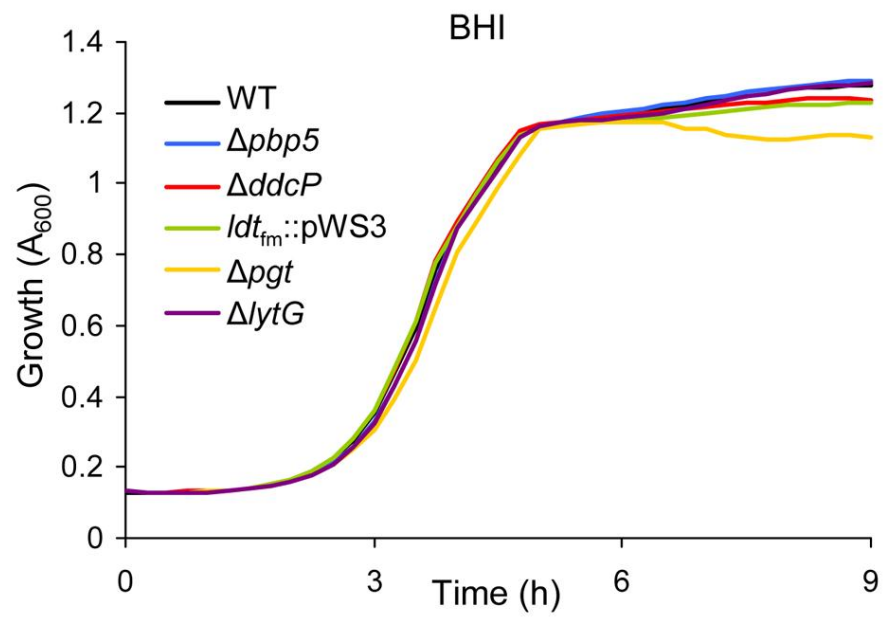

Supplement: Figure S5 — Growth curves of targeted mutants and wild-type E. faecium E1162 in BHI medium without added antibiotics. Overnight cultures of mutants and wild-type strain were inoculated at an initial cell density of OD660 0.0025 in BHI and grown at 37°C with shaking in the Bioscreen C instrument. Growth curves represent mean data from three independent experiments. (PDF) [file pgen.1002804.s005.pdf]
