# Supplementary material for: Genome-Wide Identification of Ampicillin Resistance Determinants in Enterococcus faecium
Source: PLoS Genet. 2012 Jun 28;8(6):e1002804. doi: 10.1371/journal.pgen.1002804 (PMC3386183; doi:10.1371/journal.pgen.1002804)
Supplement: Table S1 — Strains and plasmids used in this study. (DOC) [file pgen.1002804.s006.doc]

**Table S1: Strains and plasmids used in this study.**

| **Strain or plasmid** | **Relevant characteristic(s)** | **Source or reference** |
| --- | --- | --- |
| *E****.****faecium* |  |  |
| E1162 | Clinical isolate (bloodstream infection), isolated in France, 1996 | [24] |
| E745 | Strain isolated in 2000 in The Netherlands from patient feces during a VRE outbreak; resistant to vancomycin | [44] |
| E1133 | Strain isolated in 2002 in the United States of America from patient feces during a VRE outbreak; resistant to vancomycin | [44] |
| E1360 | Clinical isolate (2001, United States of America); resistant to vancomycin | [44] |
| E1391 | Clinical isolate (2000, United Kingdom) | [44] |
| Δ*ddcP* | Markerless deletion mutant of *ddcP* of E1162 | This study |
| *ldt*_fm_*::*pWS3 | Single-crossover insertional mutant of *ldt*_fm_ of E1162 | This study |
| Δ*pgt* | Markerless deletion mutant of *pgt* of E1162 | This study |
| Δ*lytG* | Markerless deletion mutant of *lytG* of E1162 | This study |
| Δ*pbp5* | Markerless deletion mutant of *pbp5* of E1162 | This study |
| Δ*ddcP+ddcP* | Complementation strain of Δ*ddcP*; Δ*ddcP* harboring pMSP3535- *ddcP* | This study |
| *ldt*_fm_*::*pWS3+ *ldt*_fm_ | Complementation strain of *ldt*_fm_*::*pWS3; *ldt*_fm_*::*pWS3 harboring pMSP3535- *ldt*_fm_ | This study |
| Δ*lytG+lytG* | Complementation strain of Δ*lytG*; Δ*lytG* harboring pMSP3535- *lytG* | This study |
| Δ*pbp5+pbp5* | Complementation strain of Δ*pbp5*; Δ*pbp5* harboring pMSP3535- *pbp5* | This study |
|  |  |  |
| *E. coli* strains |  |  |
| DH5*α* | *E. coli* host strain for routine cloning | Invitrogen |
| EC1000 | MC1000 *glgB*::*repA* | [46] |
|  |  |  |
| Plasmids |  |  |
| pMMOrf | Contains 5’ and 3’ ITR from *Himar1* | [50] |
| pAT392 | Shuttle expression vector (Gen^r^ Spc^r^*oriR*_pUC_*oriR*_pAMβ1_*oriT*_RK2_*P_2_*) | [51] |
| pCJK55 | Contains *mariner* transposase C9 induced by nisin | [47] |
| pTEX5500ts | Shuttle plasmid, gram-positive thermosensitive origin of replication; Chl^r^, Gent^r^ | [48] |
| pCJK72 | Mobilizable delivery plasmid carrying EfaMarTn transposable element; gram-positive *lacZ* gene | [47] |
| pAW068 | Transposon delivery plasmid carrying *Himar1* transpose; Gram-positive thermosensitive origin; Chl^r^, Spc^r^ | [52] |
| 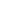pWS3 | Gram-positive thermosensitive origin; Spc^r^ | [34] |
| pZXL1 | Gram-positive *lacZ* gene form pCJK72 cloned into pTEX5500ts | This study |
| pZXL2 | Contains nisin induced *mariner* transposase C9 from pCJK55 and gram-positive thermosensitive origin of replication, Chl^r^ cassette from pZXL1 | This study |
| pZXL3 | A Gent^r^ *mariner* transposon with two outward-facing T7 promoters cloned into pZXL2 | This study |
| pZXL5 | Transposon delivery plasmid carrying nisin induced *mariner* transposase C9 and Gent^r^ *mariner* transposon with two outward-facing T7 promoters from pZXL3; Gram-positive thermosensitive origin of replication and Chl^r^ cassette from pAW068 | This study |
| pGEM-T Easy | Cloning vector | Promega |
| pGEM-ITR | ITR of *Himar1* from pMMorf ligated into pGEM-T Easy | This study |
| pGEM-Tn1 | pGEM-T Easy carrying a Gent^r^ *mariner* transposon with two outward-facing T7 promoters | This study |
| pRAB1 | *cat bla*, P_pagA_-*cre*; expression of *cre* | [56] |
| pWS3-Cre | pWS3 derivative expressing Cre in *E. faecium* | This study |
| pMSP3535 | pAMβ1 (from pIL252), ColE1 replicon, Ermr, nisRK, PnisA | [57] |
| pMSP3535- *ddcP* | Complementation plasmid for *ddcP*; pMSP3535 carrying gene *ddcP* | This study |
| pMSP3535- *ldt*_fm_ | Complementation plasmid for *ldt*_fm_; pMSP3535 carrying gene *ldt*_fm_ | This study |
| pMSP3535- *lytG* | Complementation plasmid for *lytG*; pMSP3535 carrying gene *lytG* | This study |
| pMSP3535- *pbp5* | Complementation plasmid for *pbp5*; pMSP3535 carrying gene *pbp5* | This study |
