# Supplementary material for: Genome-Wide Identification of Ampicillin Resistance Determinants in Enterococcus faecium
Source: PLoS Genet. 2012 Jun 28;8(6):e1002804. doi: 10.1371/journal.pgen.1002804 (PMC3386183; doi:10.1371/journal.pgen.1002804)
Supplement: Table S2 — Expression ratios of E. faecium E1162 genes that exhibit significant differences in expression during mid-exponential growth in BHI and BHI with 20 µg ml−1 ampicillin. (DOCX) [file pgen.1002804.s007.docx]

**Table S2: Comparative transcriptome analysis of *E. faecium* E1162 during mid-exponential growth in the presence (20 µg ml^−1^) and absence of ampicillin.**

| **LocusTag*^a^*** | **Accession code** | **Annotation** | **Expression ratio BHI+amp/BHI** |
| --- | --- | --- | --- |
| EfmE1162_0046 | ZP_06675891 | PyrR bifunctional protein | 1.9 |
| EfmE1162_0047 | ZP_06675892 | uracil permease | 2.0 |
| EfmE1162_0048 | ZP_06675893 | aspartate carbamoyltransferase | 1.9 |
| EfmE1162_0049 | ZP_06675894 | Dihydroorotase | 2.1 |
| EfmE1162_0050 | ZP_06675895 | carbamoyl-phosphate synthase, small subunit | 1.9 |
| EfmE1162_0051 | ZP_06675896 | carbamoyl-phosphate synthase, large subunit | 1.9 |
| EfmE1162_0052 | ZP_06675897 | dihydroorotate dehydrogenase electron transfer subunit | 2.0 |
| EfmE1162_0053 | ZP_06675898 | dihydroorotate dehydrogenase B, catalytic subunit | 1.9 |
| EfmE1162_0054 | ZP_06675899 | orotidine 5'-phosphate decarboxylase | 2.0 |
| EfmE1162_0055 | ZP_06675900 | orotate phosphoribosyltransferase | 2.0 |
| EfmE1162_0229 | ZP_06676074 | conserved hypothetical protein | 1.8 |
| EfmE1162_0230 | ZP_06676075 | accessory gene regulator protein B, putative | 1.7 |
| EfmE1162_0231 | ZP_06676076 | hypothetical protein | 1.8 |
| EfmE1162_0443 | ZP_06676288 | hypothetical protein | 1.7 |
| EfmE1162_0683 | ZP_06676528 | peptidoglycan-binding LysM | 0.5 |
| EfmE1162_2616 | ZP_06678315 | aggregation promoting protein | 0.4 |

*^a^* All genes exhibiting significantly different expression during mid-exponential growth (OD_660_ = 0.3) in BHI and BHI with 20 µg ml^−1^ ampicillin for all four probes per gene (Bayesian P-value <0.001) in microarray hybridizations are included.
