# Supplementary material for: Genome-Wide Identification of Ampicillin Resistance Determinants in Enterococcus faecium
Source: PLoS Genet. 2012 Jun 28;8(6):e1002804. doi: 10.1371/journal.pgen.1002804 (PMC3386183; doi:10.1371/journal.pgen.1002804)
Supplement: Table S3 — Primers used in this study. (DOC) [file pgen.1002804.s008.doc]

**Table S3: Primers used in this study^a^.**

| **Primer** | **Sequence** |
| --- | --- |
| pAT392_EheI_T7_genta_F | 5′-GGTGGGCGCC**CTCCCTATAGTGAGTCGTATTA**aaacccagcgaaccatttgaggtgataggtaag-3′ |
| pAT392_EheI_T7_genta_R | 5′-GCTCGGCGCC**CTCCCTATAGTGAGTCGTATTA**caggaaacagctatgaccatgattacgccaagc-3′ |
| pMMOrf_SacII_ITR | 5′-TCCCCGCGGTAACAGGTTGGCTGATAAGTCCCCGGTCT-3′ |
| pZXL3_BfrI_tn_F | 5′-ACGCCTTAAgaaagtcgaagggggttttt-3′ |
| pZXL3_tn_R | 5′-ttcacgaacgaaaatcaagc-3′ |
| pAW068_BfrI_cm_ori_F | 5′-ACGCCTTAAGctggcgtaatagcgaagagg-3′ |
| pAW068_BfrI_cm_ori_R | 5′-ACGCCTTAAGacgctcagtggaacgaaaac-3′ |
| pCJK72_PstI_lacZ_F | 5′-AACTGCAGGTGAGCTTGGACTAGAAAAAAACTTCACAA-3′ |
| pCJK72_KpnI_lacZ_R | 5′-GGGGTACCTTATTTTTGACACCAGACCAACTGGTAATGG-3′ |
| pZXL1_EcoRI_cm_ori_F | 5′-GGAATTCTGTACAATTGCTAGCGTACGG-3′ |
| pZXL1_EcoRI_cm_ori_R | 5′-GGAATTCGACTTCTCTACTCCTTTTAATC-3′ |
| pCJK55_EcoRI_tps_F | 5′-GGAATTCatgcatgcgtcgactctag-3′ |
| pCJK55_EcoRI_tps_R | 5′-GGAATTCaagcttcggccgatcgatg-3′ |
| genta_probe_F | 5′-ACAGAGCCTTGGGAAGATGA-3′ |
| genta_probe_R | 5′-TGCCTTAACATTTGTGGCATT-3′ |
| IPCR_HaeIII_R | 5′-ccccctgaaatccttacat-3′ |
| IPCR_HaeIII_F | 5′-aaacaggaatttatcgaaaatggt-3′ |
| ftp_tn | 5′-aaacaggaatttatcgaaaatggt-3′ |
| ftp_ddl | 5′-AAAAAGAAATCGCACCG-3′ |
| ftp_nox | 5′-TACGGTTGTAGTGACTATTTTTTC-3′ |
| ftp_esp | 5′-GGACTTGCATTAGCAAAATC-3′ |
| IPCR_AluI_F | 5′-GTTCGCTGGGTTTTAATACGACTCACT-3′ |
| IPCR_AluI_R | 5′-CAGCGAACCATTTGAGGTGATAGGT-3′ |
| pAT392_EcoRI_lox66_genta_F | 5′-GAGGGAATTC**TACCGTTCGTATAGCATACATTATACGAAGTTAT**GATAAACCCAGCGAACCATTTGAGG-3′ |
| pAT392_EcoRI_lox71_genta_R | 5′-CTCCGAATTC**TACCGTTCGTATAATGTATGCTATACGAAGTTAT**TCAATCTTTATAAGTCCTTTTATAA-3′ |
| Inst_ EcoRI_pbp5 _R | 5′-GGAATTCacctgttgtcccacgaagat-3′ |
| Inst_ XhoI_pbp5_F | 5′-CCGCTCGAGatcaaagcgattgcttcctc-3′ |
| pbp5_check | 5′-accgtctgcatctgtaatgct -3′ |
| Inst_ EcoRI_ddcP _R | 5′-GGAATTCaggctcctgcaagttcagtc-3′ |
| Inst_ XhoI_ ddcP _F | 5′-CCGCTCGAGaaaaatctctggcagcgaag-3′ |
| ddcP_check | 5′-gtgatcgtaccgacgaaaca -3′ |
| Inst_ EcoRI_ldt_fm_ _R | 5′-GGAATTCtgtttggatcgtccaactgt-3′ |
| Inst_ XhoI_ldt_fm_ _F | 5′-CCGCTCGAGcatctgaagacagctcactgaaa-3′ |
| ldtfm_check | 5′-ccacatatgctggttctcca-3′ |
| check_pWS3 | 5′-ggggattttatgcgtgagaa-3′ |
| dele_XhoI_pbp5_dn_R | 5′-CCGCTCGAGtgacgcttgtagcgattttg-3′ |
| dele_EcoRI_pbp5_dn_F | 5′-GACCACACAAGAAGCAGGAATTCtgattcagcaaccaaacgag-3′ |
| dele_EcoRI_pbp5_up_R | 5′-CAGAATTCctgcttcttgtgtggtcagg-3′ |
| dele_XmaI_pbp5_up_F | 5′-CCCCCCGGGaaaaatcgaacaggcgctta-3′ |
| check_pbp5_dn | 5′-gctgggatagctgtcagtca-3′ |
| check_pbp5_up | 5′-ggaatgacaagcaagagaagg-3′ |
| dele_XhoI_ddcP_up_F | 5′-CCGCTCGAGccacaacactatttttccatacaa-3′ |
| dele_EcoRI_ddcP_up_R | 5′-GATCGAATTCggtagaaaagaagcaaggcaaa-3′ |
| dele_EcoRI_ddcP_dn_F | 5′-GCTTCTTTTCTACCGAATTCgatcaccttggcagaagat-3′ |
| dele_ XmaI_ddcP_dn_R | 5′-CCCCCCGGGtgaaaaataggaaaaggcaaaga-3′ |
| check_ ddcP _up | 5′-gccataaacgtacccctcct-3′ |
| check_ ddcP _dn | 5′-gatcgtgatcaaggcaatca-3′ |
| dele_XhoI_pgt_dn_R | 5′-CCGCTCGAGccacattacgtaccaatggatg-3′ |
| dele_EcoRI_ pgt _dn_F | 5′-GAGGAAGCAACAATTTCAGAATTCcttctgcgattttaagtactgg-3′ |
| dele_EcoRI_ pgt _up_R | 5′-CGCAGAAGGAATTCtgaaattgttgcttcctcattg-3′ |
| dele_XmaI_ pgt up_F | 5′-CCCCCCGGGtgcaatcgaaggttcttg-3′ |
| check_ pgt _dn | 5′-acaaacagtggggcaagaag-3′ |
| check_ pgt _up | 5′-gggtagcttcaacgatttgg-3′ |
| dele_XhoI_lytG_dn_R | 5′-CCGCTCGAGtcaaaacgacccctaacgaa-3′ |
| dele_EcoRI_ lytG _dn_F | 5′-GGAAAAAGCAAAAGCGAATTCttgcaagatgcaggttatgc-3′ |
| dele_EcoRI_ lytG _up_R | 5′-GCAAGAATTCgcttttgctttttccactt-3′ |
| dele_XmaI_ lytG _up_F | 5′-CCCCCCGGGtggtctcagcttaccggtct-3′ |
| check_ lytG _dn | 5′-tttcgacgttaccgtactgat-3′ |
| check_ lytG _up | 5′-cgcattgctccagaatatga-3′ |
| comp_BamHI_ddcP_F | 5′-CGGGATCCcgcttcccgcttattagatg-3′ |
| comp_PstI_ddcP_R | 5′-AACTGCAGttttcgccaataaacgatgg-3′ |
| comp_BamHI_pbp5_F | 5′-CGGGATCCggaatgacaagcaagagaagg-3′ |
| comp_XhoI_pbp5_R | 5′-CCGCTCGAGtcattttacaaattggacagcaa-3′ |
| comp_BamHI_ldtfm_F | 5′-CGGGATCCaagtgacagcgagcaagacc-3′ |
| comp_PstI_ ldtfm_R | 5′-AACTGCAGgctgctgaaatgtaagtagcaca-3′ |
| comp_BamHI_pgt_F | 5′-CGGGATCCagaacgaatacgcacccact-3′ |
| comp_PstI_pgt_R | 5′-AACTGCAGtgcatcaagcaaacgaattt-3′ |
| comp_BamHI_lytG_F | 5′-CGGGATCCtgaaccttggcacttacgtt-3′ |
| comp_PstI_lytG_R | 5′-AACTGCAGcgaacggatcaattttccaa-3′ |
| genta_in_F | 5′-cgggaattcCAGATTGCCTTGAATATATTGAC-3′ |
| genta_in_R | 5′-gacggatccTTATTTATCACCTTTTTCATAATCA-3′ |
| ddl_1 | 5′-GAGACATTGAATATGCCTTATG-3′ |

^a^ The restriction sites are underlined, and the sequences of the T7 promoter, the *lox66* and *lox71* sites are in bold. 
